# Protein kinase a suppresses antiproliferative effect of interferon-α in hepatocellular carcinoma by activation of protein tyrosine phosphatase SHP2

**DOI:** 10.1016/j.jbc.2025.108195

**Published:** 2025-01-16

**Authors:** Yuwen Sheng, Yuan Lin, Zhe Qiang, Xiaofei Shen, Yujiao He, Lingyu Li, Sheng Li, Guolin Zhang, Fei Wang

**Affiliations:** 1 Center for Natural Products Research, Chengdu Institute of Biology, Chinese Academy of Sciences, Chengdu, China; 2 Chongqing Academy of Chinese Materia Medica, Chongqing, China; 3 Hospital of Chengdu University of Traditional Chinese Medicine, Chengdu, China; 4 Anti-infective Agent Creation Engineering Research Centre of Sichuan Province, Sichuan Industrial Institute of Antibiotics, School of Pharmacy, Chengdu University, Chengdu, China; 5 University of Chinese Academy of Sciences, Beijing, China

**Keywords:** interferons, PKA, SHP2, Cyclooxygenas 2, prostaglandin E_2_

## Abstract

Src homology-2-containing protein tyrosine phosphatase 2 (SHP2) plays a dual role in cancer initiation and progression. Identifying signals that modulate the function of SHP2 can improve current therapeutic approaches for IFN-**α/β** in HCC. We showed that cAMP-dependent PKA suppresses IFN-**α/β**–induced JAK/STAT signaling by increasing the phosphatase activity of SHP2, promoting the dissociation of SHP2 from the receptor for activated C-kinase 1 (RACK1) and binding to STAT1. Additionally, cAMP-degrading phosphodiesterase 4D (PDE4D) physically interacts with RACK1 to regulate PKA-mediated SHP2 activity and STAT1 phosphorylation. IFN-**α** activates PKA by inducing the expression of cyclooxygenase 2 (COX2) and the production of prostaglandin E_2_ (PGE_2_), which in turn stimulates the binding of SHP2 to IFNAR2 *via* RACK1. A COX inhibitor aspirin potently increases the antitumor effects of IFN-**α** in the suppression of HCC cell proliferation *in vivo*. Higher expression of COX2 and phosphorylated STAT3 is associated with poor development and prognosis in HCC patients by analyzing human HCC clinical samples. These observations suggest that a fundamental PKA/SHP2-dependent negative feedback loop acts on IFN signaling, and inhibition of this signaling by the selective COX2 inhibitors may enhance the clinical efficacy of type I IFNs in treating HCC.

Hepatocellular carcinoma (HCC)—one of the most common malignant cancers—is the third leading cause of cancer-related deaths worldwide (1) The main risk factors associated with HCC are well defined; however, it remains highly resistant to conventional systemic therapies, and the prognosis for patients with advanced HCC remains poor (2). Though type I interferons (IFNs), including IFN-α, have been used in the clinical treatment of HCC to improve overall survival and delay tumor progression, clinical trials have revealed its limited effectiveness due to the high reported toxicity (3, 4, 5). The expressions of certain genes such as hepatic IFIT3, microRNA-26, or retinoic acid–inducible gene I can help predict responses of patients to adjuvant IFN-α therapy, highlighting the effectiveness of IFN-α therapy in a subgroup of patients with HCC who responded favorably. Currently, type I IFNs (IFN-α/β) are tested in clinical trials in combination with targeted anticancer agents, chemotherapeutics, and checkpoint blockers. However, more efforts are needed to identify new combination therapy that can boost the response of IFN-α in more patients with HCC and not be not limited to the subgroup who respond well (6, 7, 8). Recently, IFN-α was found to potentiate anti-programmed cell death 1 (PD-1) efficacy in the HCC microenvironment (9, 10). However, the inactivation of the Janus kinase/signal transducer and activator of transcription (JAK/STAT) pathway is associated with acquired resistance to PD-1 blockade (11, 12). Therefore, novel therapeutic strategies are urgently necessary both to overcome IFN-α resistance and to improve the efficacy of IFN-α in patients with HCC.

The anticancer action of IFN-α is mainly attributable to its antiproliferative, antiangiogenic, and immunoregulatory activities through binding to the cell-surface receptors interferon-α receptor-1 and interferon-α receptor-2 (IFNAR1 and IFNAR2) and subsequent activation of JAK/STAT signaling pathway (13). Hence, deeply exploring regulation of JAK/STAT cascade would be conducive to enhance the anticancer action of IFN-α. Several key regulators such as suppressors of cytokine signaling proteins, protein inhibitors of activated STAT proteins, and protein tyrosine phosphatases (PTPs) negatively regulate JAK/STAT signaling (14). SHP2, encoded by *PTPN11*, is an SH2-domain—containing protein tyrosine phosphatase that is expressed in most tissues and blocks JAK tyrosine kinase activity and dephosphorylates STAT1 at both the tyrosine and serine residues to negatively regulate IFN-α—induced JAK/STAT signaling (15). It also plays a regulatory role in cell survival and proliferation by activating the RAS-ERK signaling pathway and is involved in the PD-1 immune checkpoint pathway (16). We previously showed that PKA, a classical downstream target of cAMP, interacts with IFNAR2 and regulates SHP2 through receptor for activated C-kinase 1 (RACK1), an anchoring protein that recruits STATs to IFNAR2 (17). However, it remains unclear how PKA regulation of SHP2 modulates type I IFNs-induced JAK/STAT signaling. The regulation of SHP2 activity by PKA remains controversial. For example, SHP2 can be phosphorylated by PKA, resulting in the pronounced activation of PTP activity in adrenocortical cells activated by adrenocorticotrophic hormone or in T cells (18, 19). Conversely, in cardiac myocytes, PKA can phosphorylate SHP2 at Thr73 and Ser189 through PKA-anchoring protein (AKAP)-Lbc, thereby inhibiting PTP activity and disrupting its binding to tyrosine-phosphorylated ligands (20). Therefore, the discrepancy in SHP2 activity modulated by PKA may be tissue-specific, a possible new mechanism in the determination of JAK/STAT pathway tissue specificity. cAMP regulates a wide range of important biological processes through PKA as a key intracellular messenger of numerous hormones, cytokines, and neurotransmitters. The amplitude and duration of cAMP signaling depend on the activity of cAMP-degrading phosphodiesterases (PDEs), such as PDE4D, which often form a signalosome complex with AKAPs and PKA in specific subcellular structures to spatiotemporally confine cAMP signaling. Therefore, elucidating the mechanism of the compartmentalized modulation of SHP2 activity by PKA/PDE-regulated cAMP signaling would provide a new fundamental understanding of the JAK/STAT pathway. Interestingly, we previously identified RACK1 as a new AKAP protein that can anchor PKA and PDE4D to IFNAR2 (17); however, the function and mechanism of this PKA/PDE4D/RACK-1 signalosome in the regulation of type I IFNs in the JAK/STAT signaling pathway remains unclear. SHP2 plays a key role in RAS-driven cancers, and its targeting has been validated in clinical trials as a promising strategy for cancer immunotherapy using several allosteric inhibitors (21). Therefore, understanding the crosstalk between cAMP and JAK/STAT would be helpful for developing new therapeutics to enhance the efficacy of type I IFNs and SHP2 inhibitors for cancer treatment.

Prostaglandin E_2_ (PGE_2_), a well-known pro-inflammatory cytokine, is overexpressed in various human malignancies including HCC, which binds to prostaglandin E receptors in HCC cells to promote the proliferation and migration of liver cancer cells through multiple pathways related with PKA (22, 23). Cyclooxygenase 2 (COX2), a rate-limiting enzyme responsible for PGE_2_ biosynthesis, is not expressed in most normal tissue but is rapidly induced in response to several tumor promotors and cytokines mainly through NF-κB and STAT3 signalings (24). Increasing evidence has highlighted the enhancement of high expression COX2 on cell proliferation, apoptosis inhibition, immune evasion, and chemotherapy resistance, which contributes to hepatocarcinogenesis and reduced survival of patients with HCC (25, 26). Experimentally, some COX2 inhibitors such as celecoxib and aspirin have been shown to inhibit human HCC cells because of their antiproliferative and pro-apoptotic effects (27, 28, 29). However, efforts to develop new therapeutic strategies targeting this pathway with the single use of antagonists of the prostaglandin E receptor or COX2 are impaired by the side effects associated with abundant functions of PGE_2_ in physiological and pathological processes. Therefore, it is important to explore the combined use of drugs targeting the COX2/PGE_2_ axis with other antitumor drugs will show great potential in HCC. Interestingly, type I IFNs have been reported to induce COX2 expression in human hepatoma cells and synergistically induce apoptosis with COX2 inhibitors in human hepatoma cells *in vitro* and *in vivo* (30). However, crosstalk between the COX2/PGE_2_ and JAK/STAT pathways remains unclear. In the present study, we identified a novel negative feedback loop of type I IFNs in that IFN-α stimulates the expression of COX2 through the activation of STAT3/NF-κB signaling, leading to the increased production of PGE_2_ which activates PKA to promote the PTP activity of SHP2 in IFNAR2-bound RACK1/PKA/PDE4D signalosome, ultimately attenuating JAK/STAT pathway signaling. The COX2 inhibitors significantly enhanced IFN-α–mediated JAK/STAT pathway activation and antiproliferation on HCC cells *in vitro* and *in vivo*. These findings provide new insights on the understanding of the cause of less effectiveness of IFN-α therapy in HCC and helpful to develop new combination therapeutic strategy for treating cancers with IFN-α in clinical practice.

## Results

### PKA suppresses JAK/STAT pathway signaling *via* SHP2

**Figure 1:**
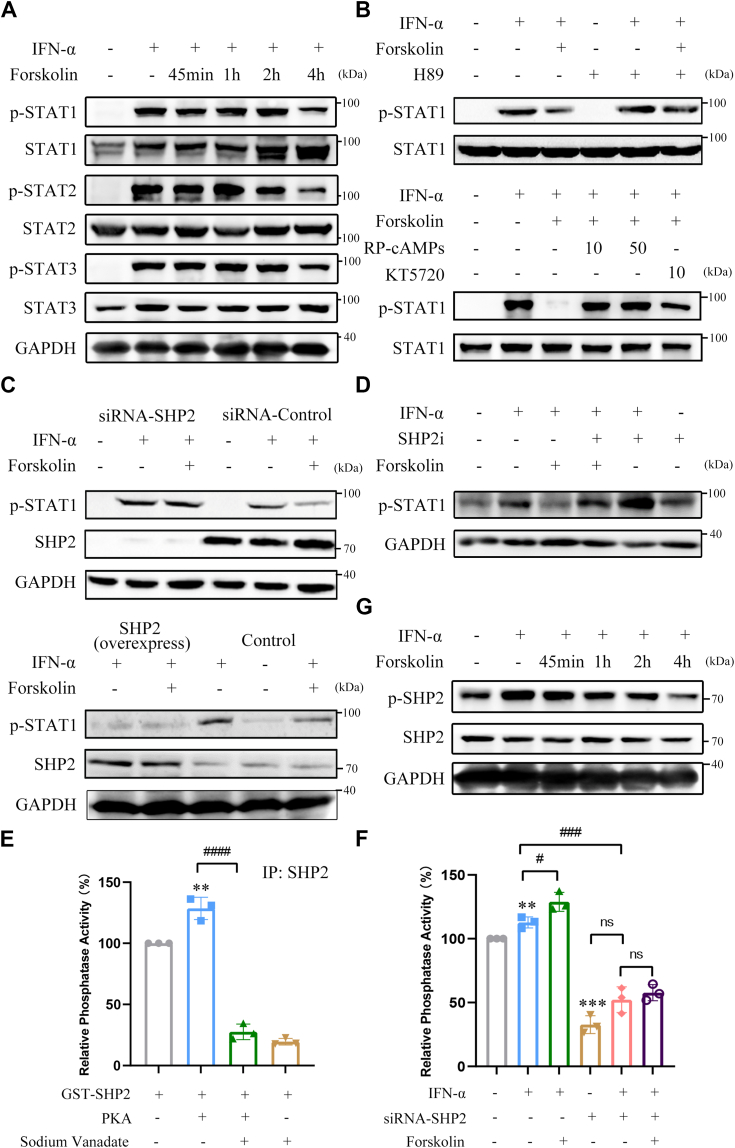
**SHP2 mediates the inhibitory effect of cAMP-dependent PKA on JAK/STAT signaling pathway.***A and G*, Huh-7 cells were pretreated with DMSO or 50 μM forskolin for 45 min, 1 h, 2 h, 4 h, respectively, and then treated with IFN-α for 30 min. Cell lysates were immunoblotted with phospho-STAT1 (Tyr701), phospho-STAT2 (Tyr690), phospho-STAT3 (Tyr705), STAT1, STAT2, STAT3 (*A*), and phospho-SHP2 (Tyr690), SHP2 (*G*) antibodies. GAPDH was used as a loading control. *B* and *D*, HEK293A cells were pretreated with DMSO, 10 μM H89, 10 or 50 μM RP-cAMPs or 10 μM KT5720 (*B*), and 200 μM SHP2 inhibitor (SHP2i) (*D*) for 1 h and then with forskolin for 45 min before treatment with IFN-α for 30 min. Cell lysates were immunoblotted with phospho-STAT1 (Tyr701) and STAT1 antibodies. *C*, HEK293A cells were transfected with siRNAcon (50 nM), SHP2 siRNA001 (50 nM) (the *upper* panel), or pCMV3-SHP2 plasmids (the *lower* panel). After 72 h, cells were pretreated with forskolin for 45 min and then incubated with IFN-α for 30 min. Cell lysates were immunoblotted with phospho-STAT1 (Tyr701), SHP2, and GAPDH antibodies. *E*, purified recombinant GST-SHP2 was incubated with the PKA catalytic subunit or sodium vanadate for 30 min in 100 μl reaction buffer containing 10 μM DIFMUP and then detected with a microplate reader (excitation, 355 nm; emission, 460 nm). ∗∗*p* < 0.01 *versus* GST-SHP2 group, ####*p* < 0.0001 *versus* GST-SHP2 + PKA group (unpaired two-tailed Student’s *t* test). *F*, Huh-7 cells were transfected with siRNAcon (50 nM), SHP2 siRNA001 (50 nM). After 72 h, cells were pretreated with forskolin for 4 h and then incubated with IFN-α for 30 min. The SHP2 proteins were co-immunoblotted using SHP2 antibodies from the cell lysates and the activity of SHP2 proteins in each group was detected using microplate reader (excitation, 355 nm; emission, 460 nm). ∗∗*p* < 0.01, ∗∗∗*p* < 0.001 *versus* control group, #*p* < 0.05 and ###*p* < 0.001 *versus* IFN-α treatment group; ns, nonsignificant (unpaired two-tailed Student’s *t* test). IFN-α, 5000 U/ml; IFN-β, 1000 U/ml; forskolin, 50 μM; sodium vanadate, 50 μM; PKA catalytic subunit, 25 U/μl. All experiments were conducted with three independent replicates and the results of representative data are shown. The data are presented as the mean ± SD from three independent experiments.

To determine whether cAMP-dependent PKA influences IFN-α–mediated JAK/STAT signaling in HCC cells, different cAMP elevators and PKA inhibitors were used. Forskolin (FSK), a well-known adenylate cyclase agonist for stimulating intracellular cAMP production, significantly attenuated IFN-α/β–induced phosphorylation of STAT1, STAT2, and STAT3 in Huh-7 and HCCLM3 HCC cells (Fig. 1*A* and Fig. S1*A*). FSK also suppressed IFN-α/β–induced phosphorylation of STAT1 in HEK293A cells (Fig. S1*B*). However, treatment with actinomycin D and cycloheximide did not further enhance the inhibitory effect of FSK on STAT1 phosphorylation in Huh-7 cells, indicating that FSK directly suppressed STAT1 phosphorylation, rather than modulating it indirectly at the transcriptional or translational level (Fig. S1, *C* and *D*). Furthermore, treatment with PKA inhibitors such as H89, RP-cAMPs, or KT-5720 significantly reversed the inhibitory effect of FSK on IFN-α–induced phosphorylation in HEK293A and Huh-7 cells (Fig. 1*B* and Fig. S1, *E–I*). The mRNA expression of *PKR* and *2′,5′-OAS*, two IFN-α responsive genes, were markedly decreased by FSK or PGE_2_ treatment; this effect can be reversed by pretreatment with H89 (Fig. S1, *J* and *K*). These results suggest that cAMP-dependent PKA suppresses IFN-α–mediated signaling and action. Previously, we found that PKA interacts with IFNAR2 and may inhibit the activation of the JAK/STAT pathway *via* regulation of SHP2, although the mechanism remains unknown (17). We thus further investigated whether the attenuation of PKA on IFN-α–induced STAT1 phosphorylation was mediated by SHP2 protein. As expected, SHP2 knockdown significantly attenuated the FSK-induced inhibitory effect on IFN-α–induced STAT1 phosphorylation in HEK293A and Huh-7 cells (Fig. 1*C*, the upper panel, Fig. S1, *L* and *M*). Consistent with that, SHP2 inhibitor such as sodium vanadate and SHP099 not only increased the IFN-α/β–induced STAT1 phosphorylation but also significantly attenuated the inhibitory effect of FSK on STAT1 phosphorylation (Fig. 1*D* and Fig. S1, *O–R*). Conversely, overexpression of SHP2 further potentiated the inhibitory effect of FSK on IFN-α–induced phosphorylation of STAT1 (Fig. 1C, the lower panel, and Fig. S1*N*). Next, we assessed whether PKA altered the SHP2 activity. As shown in Fig. 1*E*, the PTP activity of the recombinant SHP2 protein was remarkably increased by incubation with PKA catalytic subunits, which was significantly attenuated by sodium vanadate, a pan-PTP inhibitor. Interestingly, IFN-α treatment also increased the PTP activity of SHP2, which was further enhanced by FSK treatment and abolished by the knockdown of SHP2 and the SHP2 inhibitor in Huh-7 cells (Fig. 1*F* and Fig. S1, *S* and *T*). However, FSK significantly inhibited IFN-α–induced phosphorylation of SHP2 at Tyr690 (Fig. 1*G*). Overall, these results suggest that PKA enhances SHP2 activity, thereby negatively regulating IFN-α–induced JAK-STAT signaling.

### PKA promotes the binding of SHP2 to STAT1 at IFNAR2

**Figure 2:**
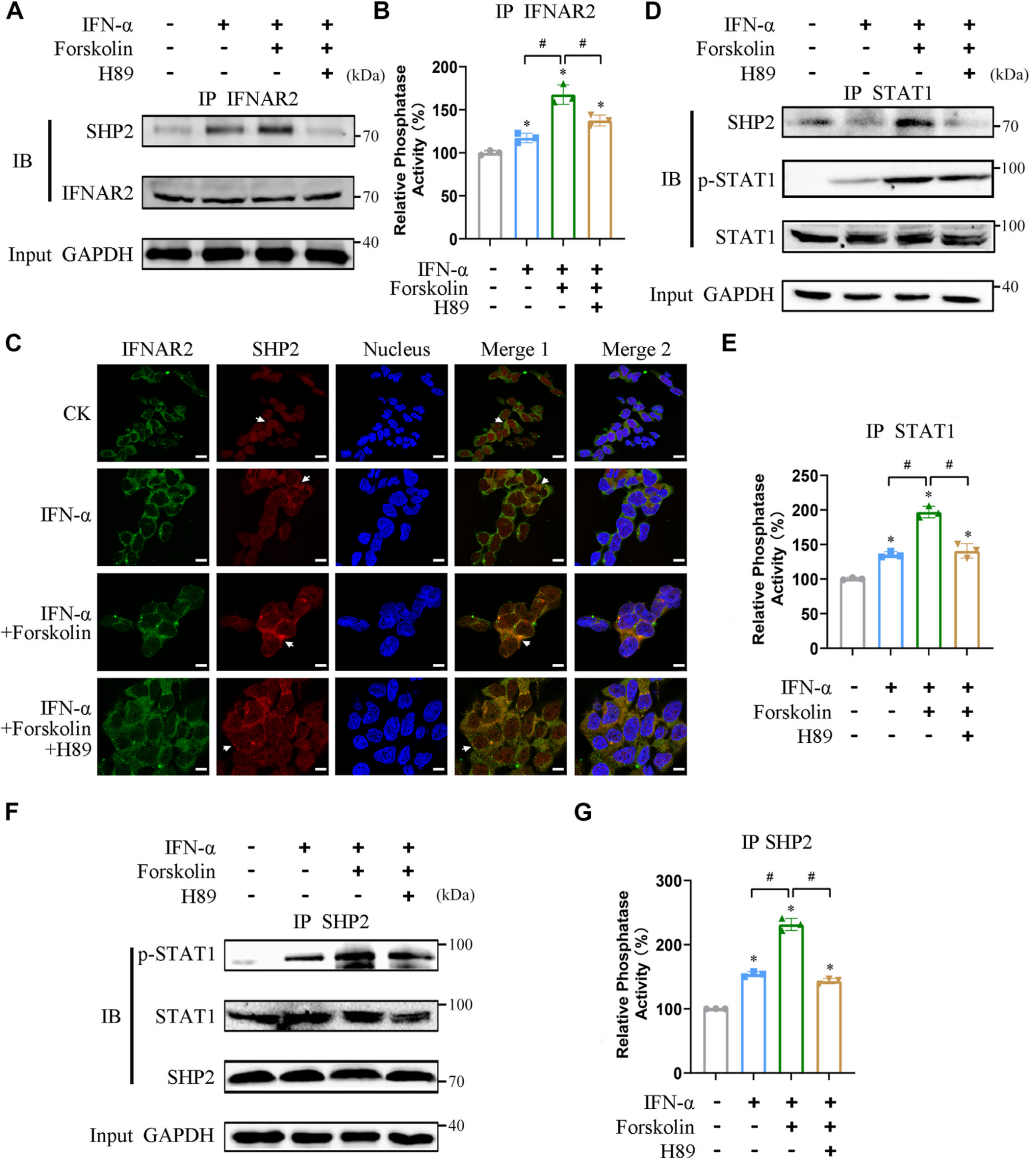
**PKA promotes SHP2 interaction with STAT1.** HEK293A cells were pretreated with DMSO or H89 for 1 h and forskolin for 45 min before treatment with IFN-α for 30 min. Cell lysates were immunoprecipitated with IFNAR2 antibody, and the co-immunoprecipitation (Co-IP) products were divided into two parts. *A*, one sample was immunoblotted with an SHP2 antibody. IFNAR2 was used as a loading control. *B*, another sample was detected in another phosphatase activity of SHP2. ∗*p* < 0.05 *versus* control group, #*p* < 0.05 *versus* IFN-α + forskolin treatment group (unpaired two-tailed Student’s *t* test). *C*, HEK293A cells were pretreated with DMSO or H89 for 1 h and then with forskolin for 45 min before treatment with IFN-α for 30 min. Colocalization of IFNAR2/SHP2 was analyzed by immunofluorescence. IFNAR2 and SHP2 staining are shown in *green* and *red*, respectively, and *arrows* indicate the localization of IFNAR2 and SHP2 following different drug treatments. Nuclei were stained with DAPI. Merge 1 was merged with IFNAR2 and SHP2, and the *arrows* indicate the regions of apparent colocalization (*yellow*) with different drug treatments. Merge 2 was merged with IFNAR2, SHP2, and the nuclei. Scale bar represents 10 μm. *D* and *E*, cell lysates were immunoprecipitated with STAT1 antibody, and the Co-IP products were divided into two parts. One sample was immunoblotted with SHP2 and phospho-STAT1 (Tyr701) antibodies. STAT1 was used as a loading control (*D*). Another sample was used to detect the phosphatase activity of SHP2 (*E*). ∗*p* < 0.05 *versus* control group, #*p* < 0.05 *versus* IFN-α + forskolin treatment group (unpaired two-tailed Student’s *t* test). *F and G*, cell lysates were immunoprecipitated with SHP2 antibody, and the Co-IP products were divided into two parts. One sample was immunoblotted using antibodies against STAT1 and phospho-STAT1 (Tyr701). SHP2 antibody staining was used as a loading control (*F*). The other sample was used to detect the phosphatase activity of SHP2 (*G*). ∗*p* < 0.05 *versus* control group, #*p* < 0.05 *versus* IFN-α + forskolin treatment group (unpaired two-tailed Student’s *t* test). CK, DMSO, 0.1%; IFN-α, 5000 U/ml; H89, 10 μM; forskolin, 50 μM. All experiments were conducted with three independent replicates and the results of representative data are shown. The data are presented as the mean ± SD from three independent experiments.

To further investigate the role of PKA in the negative regulation of IFN-α signaling, we evaluated whether PKA alters the interaction of SHP2 to STAT1 at IFNAR2. Co-immunoprecipitation (Co-IP) using IFNAR2 antibody showed that FSK treatment stimulated IFN-α-induced binding of SHP2 to IFNAR2, which could be inhibited by the PKA inhibitor H89 (Fig. 2*A* and Fig. S2*A*). Consistent with this, FSK treatment promoted the PTP activity of SHP2 in IFNAR2, which was attenuated by H89 (Fig. 2*B* and Fig. S2*B*). Additionally, FSK also enhanced the dephosphorylation of STAT1 at IFNAR2 (Fig. S2*C*). This finding was further supported by immunofluorescence experiments, in which FSK treatment simulated the colocalization of SHP2 with IFNAR2, whereas it was abolished by H89 treatment (Fig. 2C and Fig. S2*D*). Moreover, examination of co-immunoprecipitates with the STAT1 antibody showed that FSK treatment stimulated the binding of SHP2 to STAT1, which was abolished by H89 (Fig. 2*D* and Fig. S2*E*). Similarly, the PTP activity of SHP2 in STAT1 was significantly enhanced by FSK treatment and was abolished by H89 (Fig. 2*E* and Fig. S2*F*). Interestingly, more phosphorylated STAT1 interacted with SHP2 upon treatment with FSK (Fig. 2*D* and Fig. S2*E*). This was further supported by the observation that FSK treatment stimulated the binding of phosphorylated STAT1 to SHP2 at 45 min, which was also abolished by H89 (Fig. 2*F* and Fig. S2*G*). Similarly, PTP activity in SHP2 immunoprecipitants was enhanced by FSK and abolished by H89 at 45 min (Fig. 2*G* and Fig. S2*H*). Furthermore, as the FSK treatment was prolonged, the phosphorylation level of STAT1 progressively decreased at 4 and 6 h (Fig. S2*I*). These findings suggested the promoting effect of PKA in the binding of SHP2 to STAT1 at IFNAR2, as well as the local activity of SHP2, which negatively regulates IFN-α–induced signaling.

### PKA promotes the dissociation of SHP2 from RACK1

**Figure 3:**
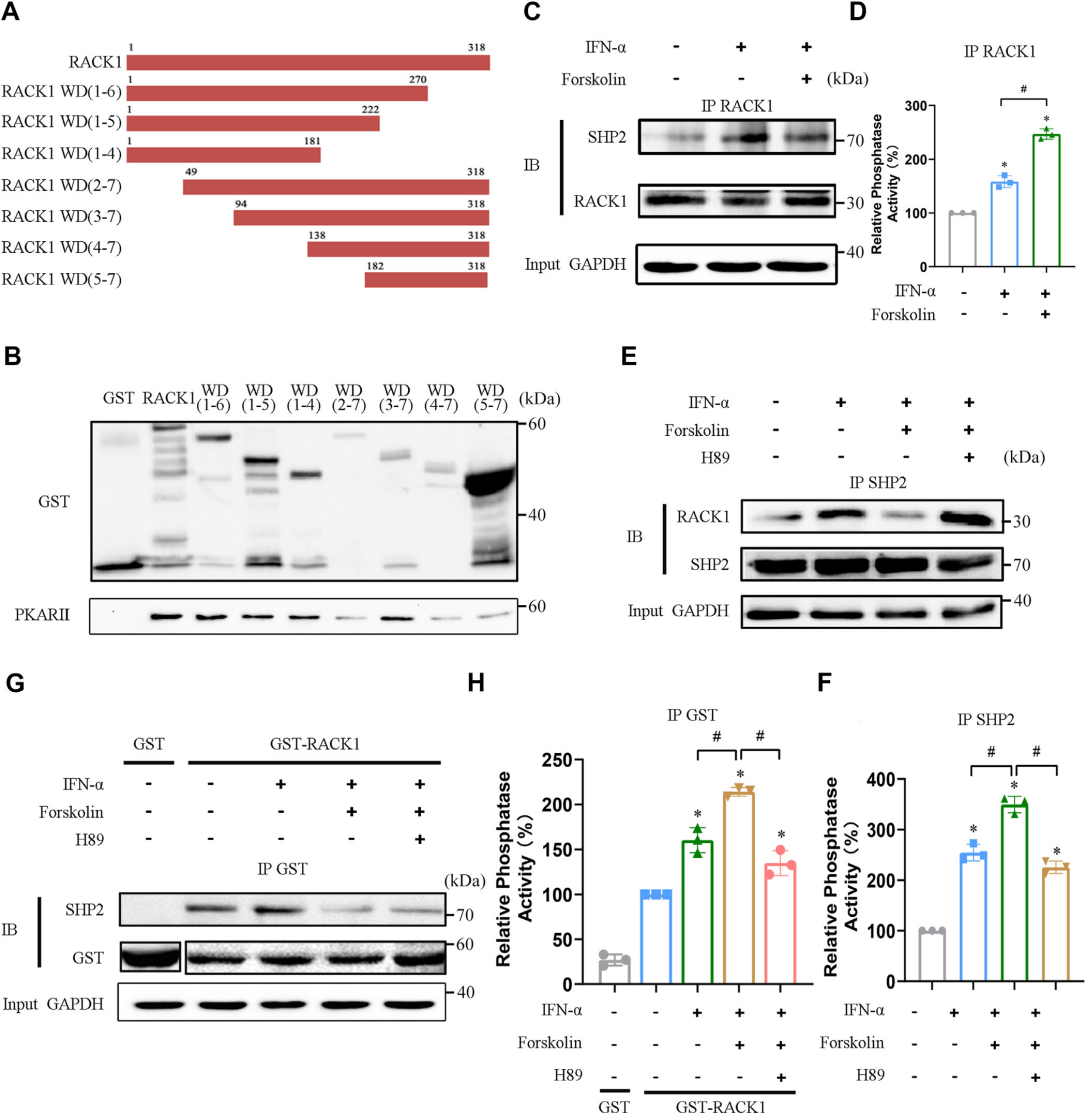
**PKA promotes the dissociation of SHP2 from RACK1.***A*, schematic diagram illustrating truncated RACK1 proteins. *B*, GST or GST-RACK1 truncation fusion proteins were incubated with recombinant expressed PKA RIIα respectively and immunoblotted with PKA RII antibody. GST antibody staining was used as a loading control. *C* and *D*, HEK293A cells were pretreated with DMSO or forskolin for 45 min. After that the cells were treated with IFN-α for 30 min. Cell lysates were immunoprecipitated with the RACK1 antibody, and the Co-IP products were divided into two parts. One sample was immunoblotted using an SHP2 antibody. RACK1 antibody was used as a loading control (*C*). The other sample was used to detect the phosphatase activity of SHP2 (*D*). ∗*p* < 0.05 *versus* control group, #*p* < 0.05 *versus* IFN-α treatment group (unpaired two-tailed Student’s *t* test). *E* and *F*, HEK293A cells were pretreated with DMSO or H89 for 1 h, and then with forskolin for 45 min. After that, the cells were treated with IFN-α for 30 min. Cell lysates were immunoprecipitated with the SHP2 antibody, and the Co-IP products were divided into two parts. One sample was immunoblotted with the RACK1 antibody. SHP2 antibody was used as a loading control (*E*). The other sample was used to detect the phosphatase activity of SHP2 (*F*). ∗*p* < 0.05 *versus* control group, #*p* < 0.05 *versus* IFN-α + forskolin treatment group (unpaired two-tailed Student’s *t* test). *G* and *H,* GST or GST-RACK1 fusion proteins were incubated with the indicated cell lysates. The pull-down products were divided into two parts. One sample was immunoblotted using an SHP2 antibody. GST antibody staining was used as a loading control (*G*). The other sample was used to detect the phosphatase activity of SHP2 (*H*). ∗*p* < 0.05 *versus* control group, #*p* < 0.05 *versus* IFN-α + forskolin treatment group (unpaired two-tailed Student’s *t* test). IFN-α, 5000 U/ml; H89, 10 μM; forskolin. 50 μM. All experiments were conducted with three independent replicates and the results of representative data are shown. The data are presented as the mean ± SD from three independent experiments.

Previously, we showed that PKA interacts with IFNAR2 through RACK1; however, the domain of RACK1 that is required for this interaction remains unclear. RACK1 consists of seven WD domains (Fig. S3*A*); therefore, a variety of truncated RACK1 proteins were examined for interaction with PKA RII protein using a pull down assay (Fig. 3*A*). Truncation of the WD-3 and WD-4 domains of RACK1 significantly attenuated its interaction with PKA RII, whereas the other truncated domains of RACK-1 had no obvious effect on the interaction between RACK1 and PKA RII (Fig. 3*B* and Fig. S3*B*). To examine the effect of PKA activation on the interaction between RACK1 and SHP2, we performed Co-IP using anti-RACK1 antibody. As shown in Fig. 3*C*, IFN-α treatment increased the binding of SHP2 to RACK1, whereas FSK treatment significantly inhibited the binding of SHP2 with RACK1. Additionally, IFN-α–induced increase in the local activity of SHP2 in the RACK1 antibody immunoprecipitant was further boosted by FSK (Fig. 3*D*). This was further supported by the observation that FSK treatment suppressed the binding of RACK1 to SHP2, which was also abolished by H89 (Fig. S3*C*). Similarly, PTP activity in SHP2 immunoprecipitants was enhanced by FSK and abolished by H89 (Fig. S3*D*). In addition, recombinant RACK1 was used to examine the interaction between RACK1 and SHP2. Similarly, FSK treatment also significantly inhibited the IFN-α–induced binding of SHP2 to RACK1, which was partially rescued by H89 treatment (Fig. 3*E*). Moreover, IFN-α–induced increase in the local activity of SHP2 in recombinant-expressed RACK1 protein was further boosted by FSK but abolished by H89 treatment (Fig. 3*F*). Co-IP was performed using anti-SHP2 antibody. As shown in Fig. 3*G*, IFN-α treatment increased the binding of RACK1 to SHP2, which was significantly inhibited by FSK treatment and rescued by H89 treatment. IFN-α–induced increase in the local activity of SHP2 in the SHP2 antibody immunoprecipitant was further boosted by FSK but abolished by H89 treatment (Fig. 3*H*). These results suggest that PKA activation can promote the dissociation of SHP2 from RACK1, thereby boosting the binding of SHP2 to STAT1 at IFNAR2.

### PKA phosphorylates SHP2 at Ser234 to enhance its activity

**Figure 4:**
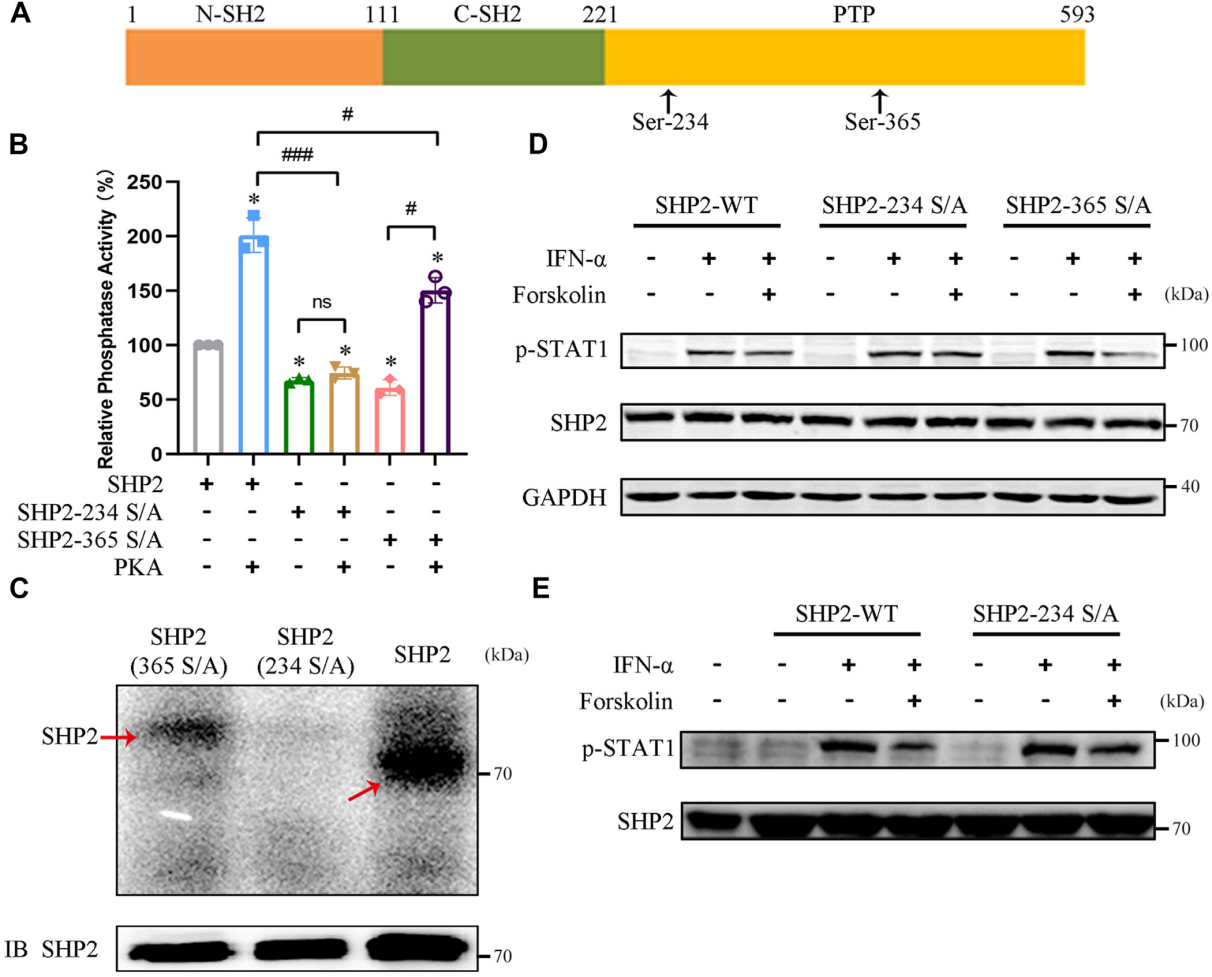
**PKA phosphorylates SHP2 and promotes its activity.***A*, schematic diagram of the SHP2 mutants. *B*, purified recombinant GST-SHP2, GST-SHP2^S234A^ mutant, and GST-SHP2^S365A^ mutant were pretreated with the PKA catalytic subunit for 30 min, and the phosphatase activity of SHP2 was examined. ∗*p* < 0.05 *versus* SHP2 group, #*p* < 0.05 *versus* SHP2-365 S/A group, and #*p* < 0.05, ###*p* < 0.001 *versus* SHP2 + PKA group; ns, nonsignificant (unpaired two-tailed Student’s *t* test). *C*, purified recombinant GST-SHP2, GST-SHP2^S234A^, and GST-SHP2^S365A^ were pretreated with the PKA catalytic subunit in the presence of γ-32P ATP for 30 min. The samples were separated by SDS-PAGE, and phosphorylated SHP2 was detected by autoradiography. SHP2 was used as a loading control. *D*, HEK293A cells were transiently transfected with pCMV-SHP2, pCMV-SHP2^S234A^, or pCMV-SHP2^S365A^ plasmids. After 48 h, cells were pretreated with DMSO or forskolin for 45 min and then treated with IFN-α for 30 min. Cell lysates were immunoblotted using antibodies against phospho-STAT1 (Tyr701) or SHP2. GAPDH was used as the loading control. *E*, Huh-7 cells transiently transfected with pCMV-SHP2 or pCMV-SHP2^S234A^. After 48 h, the cells were pretreated with DMSO or forskolin for 4 h and then treated with IFN-α for 30 min. Cell lysates were immunoblotted using antibodies against phospho-STAT1 (Tyr701) or SHP2. IFN-α, 5000 U/ml; forskolin. 50 μM. All experiments were conducted with three independent replicates and the results of representative data are shown. The data are presented as the mean ± SD from three independent experiments.

We investigated whether PKA enhanced SHP2 activity through direct phosphorylation. We first identified two conserved PKA phosphorylation sites, Ser234 and Ser365, in SHP2 using GPS2.1 and pkaPS bioinformatics methods (Fig. 4*A*) (31, 32). Interestingly, these two amino acids in SHP2 are conserved from *Xenopus laevis* to mammalian species, including mice, monkeys, and humans (Fig. S4*A*). To confirm whether these two serine residues are the phosphorylation sites of PKA, we mutated each serine residue to alanine. The catalytic subunit of PKA stimulated the PTP activity of the WT recombinant SHP2 protein *in vitro*; however, mutations of Ser234 to alanine (S234A) and Ser365 to alanine (S365A) both caused obvious inhibition of the PKA-mediated elevated PTP activity of SHP2 (Fig. 4*B*). *In vitro* phosphorylation assays showed that SHP2 phosphorylation by PKA was strongly reduced by S234A or S365A mutations (Fig. 4*C*), suggesting that phosphorylation probably occurs at S234A or S365A. We next investigated the functional requirement of Ser234 and Ser365 of SHP2 for the PKA-mediated reduction in STAT1 phosphorylation. Similar to WT SHP2, the phosphorylation of STAT1 was still reduced by FSK in IFN-α–treated HEK293A cells overexpressing SHP2^S365A^ protein but not SHP2^S234A^ protein (Fig. 4*D*). This was further corroborated in Huh-7 cells overexpressing WT SHP2 protein, where FSK was shown to promote the phosphorylation of SHP2 at Ser234. The SHP2^S234A^ mutation abolished the phosphorylation of SHP2 at Ser234 induced by FSK, as well as the inhibitory effect on IFN-α–induced STAT1 phosphorylation (Fig. 4*E* and Fig. S4*B*). Furthermore, Co-IP with an IFNAR2 antibody showed that overexpression of WT SHP2 further enhanced the interaction between SHP2 and IFNAR2, whereas the SHP2^S234A^ mutation failed to increase this interaction (Fig. S4*C*). These results suggested that PKA specifically phosphorylates SHP2 at Ser234 to enhance PTP activity, thereby disrupting the interaction between SHP2 and IFNAR2, and ultimately attenuating type I IFNs-induced activation of JAK/STAT signaling.

### PDE4D regulates SHP2 activity *via* RACK1

**Figure 5:**
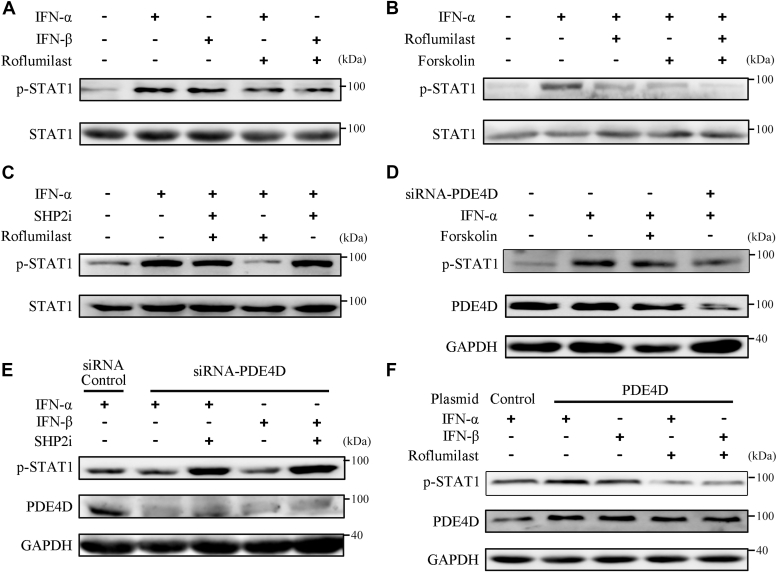
**PDE4D regulates SHP2 activity *via* RACK1.***A*, Huh-7 cells were pretreated with DMSO or 1 μM Roflumilast for 1 h before treatment with IFN-α/β for 30 min. Cell lysates were immunoblotted using antibodies against phospho-STAT1 (Tyr701) and STAT1. *B*, Huh-7 cells were pretreated with DMSO or 1 μM Roflumilast for 1 h and then with forskolin for 45 min before treatment with IFN-α for 30 min. Cell lysates were immunoblotted using antibodies against phospho-STAT1 (Tyr701) and STAT1. *C*, Huh-7 cells were pretreated with DMSO or 200 μM SHP2 inhibitor (SHP2i) or 1 μM Roflumilast for 1 h before treatment with IFN-α (IFN-α, 5000 U/ml) for 30 min. Cell lysates were immunoblotted using antibodies against phospho-STAT1 (Tyr701) and STAT1. *D*, Huh-7 cells were transfected with control siRNA (50 nM) or siRNA-PDE4D (50 nM). After 72 h, the cells were pretreated with DMSO or forskolin for 45 min before treatment with IFN-α for 30 min. The cell lysates were immunoblotted using antibodies against phospho-STAT1 (Tyr701), STAT1, and GAPDH. *E*, Huh-7 cells were transfected with control siRNA (50 nM) or siRNA-PDE4D (50 nM). After 72 h, the cells were pretreated with DMSO or 200 μM SHP2 inhibitor (SHP2i) for 1 h before treatment with IFN-α/β for 30 min. The cell lysates were immunoblotted using antibodies against phospho-STAT1 (Tyr701), PDE4D, and GAPDH. *F*, Huh-7 cells were transiently transfected with the pCMV-PDE4D plasmids. After 48 h, cells were pretreated with DMSO or 1 μM Roflumilast for 1 h before treatment with IFN-α/β for 30 min. The cell lysates were immunoblotted using antibodies against phospho-STAT1 (Tyr701), PDE4D, and GAPDH. IFN-α, 5000 U/ml; IFN-β,1000 U/ml. All experiments were conducted with three independent replicates and the results of representative data are shown.

Compartmentalized cAMP signaling is mediated by distinct PDE isoforms that dynamically hydrolyze cAMP. Therefore, we examined whether cAMP-hydrolyzing PDE4D, which we previously found to interact with IFNAR2, participates in PKA-mediated negative regulation of JAK/STAT signaling (17). Roflumilast, a PDE4D inhibitor, significantly attenuated STAT1 phosphorylation induced by IFN-α/β in Huh-7 cells (Fig. 5*A*). Roflumilast further enhanced the inhibitory effect of FSK in IFN-α–induced STAT1 phosphorylation (Fig. 5*B* and Fig. S5*A*). Rolipram, another specific PDE4D inhibitor, also enhance the inhibitory effect of FSK on IFN-β–induced STAT1 phosphorylation (Fig. S5*B*). SHP2 inhibitor treatment attenuated the suppressive effect of roflumilast on IFN-α/β–induced STAT1 phosphorylation (Fig. 5*C* and Fig. S5*C*). Knockdown of PDE4D significantly suppressed IFN-α/β–induced phosphorylation of STAT1 (Fig. 5*D*). Furthermore, the suppressive effect of PDE4D knockdown on STAT1 phosphorylation was significantly reversed by the SHP2 inhibitor treatment (Fig. 5*E*). Consistent with this, the knockdown of PDE4D also stimulated the activity of SHP2 (Fig. S5*D*). Moreover, overexpression of PDE4D enhanced the phosphorylation of STAT1 induced by IFN-α/β, which could be further suppressed by roflumilast (Fig. 5*F*). Furthermore, the recombinant PDE4D protein directly interacted with the recombinant GST-RACK1 protein, but not with GST alone (Fig. S5*E*). These results suggested that PDE4D interacts with RACK1 to regulate SHP2 activity.

### Transcriptome analysis of PKA activation in HCC cells

**Figure 6:**
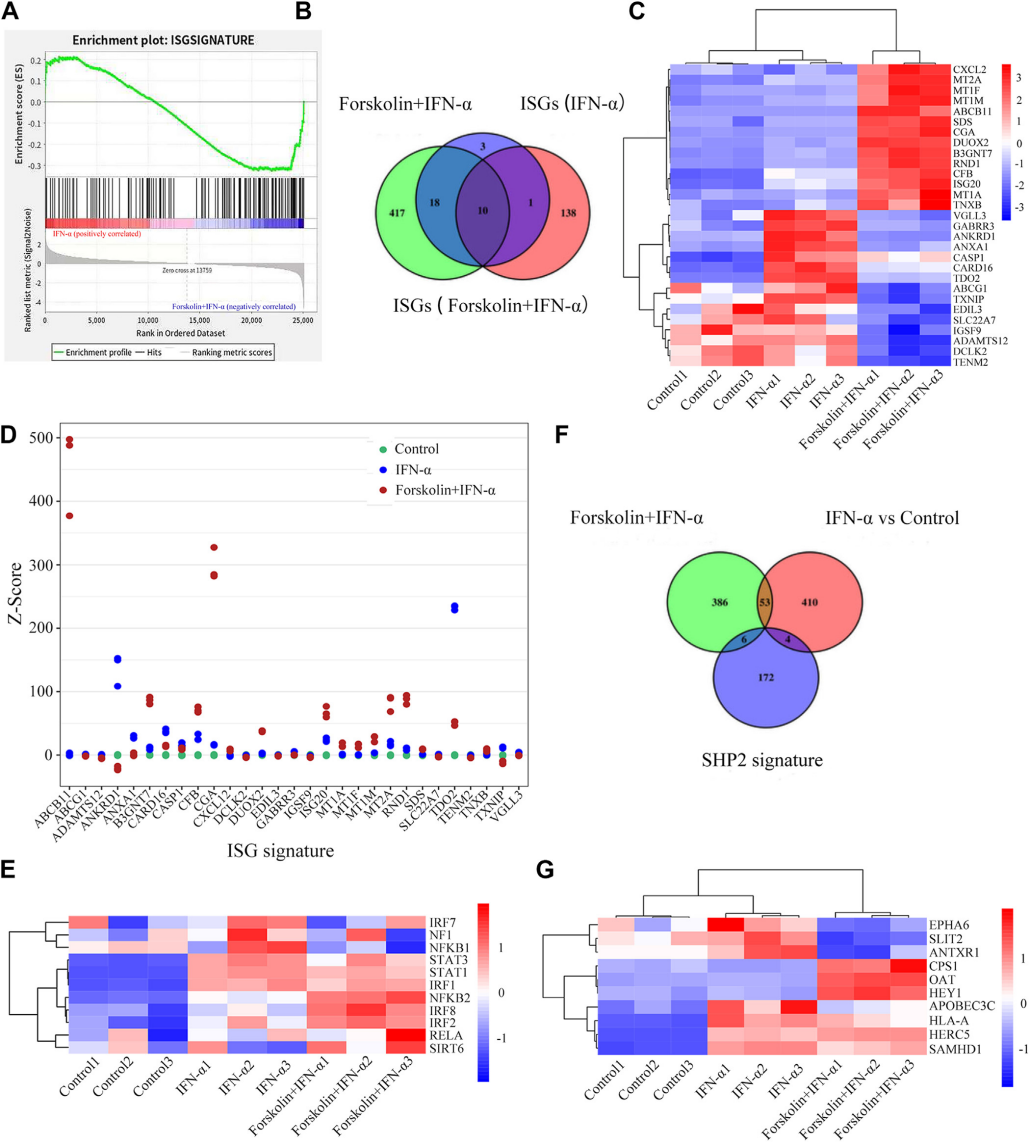
**PAK regulates SHP2 signatures and inhibits IFN-stimulated genes (ISGs) transcription.***A*, gene set enrichment analysis (GSEA) of the ISGs. Rank statistics (bottom; y-axis) and normalized enrichment scores (top; y-axis) indicate downregulation and upregulation, respectively. Normalized enrichment score (NES) = −1.34, *p* < 0.05, FDR *q* = 0.034. *B*, twenty nine common ISGs expressed in forskolin + IFN-α (differentially expressed genes in the forskolin + IFN-α treatment group compared to the IFN-α treatment group), ISGs (IFN-α) (differentially expressed ISGs in the IFN-α treatment group), and ISGs (forskolin + IFN-α) (differentially expressed ISGs in the forskolin and IFN-α treatment group) were selected for further analysis. *C*, heat map hierarchical clustering displaying differentially expressed genes in ISGs with *p* < 0.05. *D*, Z-score of the expression data from twenty nine ISGs in control, IFN-α, and forskolin + IFN-α groups. *E*, heat map hierarchical clustering displaying the differentially expressed genes of transcription factors related to ISGs regulation. *F*, Venn diagram showing the overlap of differential expressed genes among the IFN-α, forskolin + IFN-α groups, and total SHP2 signature. *G*, heat map hierarchical clustering displaying the differentially expressed genes in the 10 SHP2 signature. (n = 3, each group).

To further explore the regulatory role of PKA in IFN-α signaling, we evaluated the expression profile of interferon-stimulated genes (ISGs) using transcriptomics. We first obtained type I IFN-associated ISGs based on the interferome database and screened 158 ISGs by differential gene expression analysis of FSK plus IFN-α and IFN-α groups. Gene Set Enrichment Analysis (GSEA) showed that the addition of FSK significantly negatively regulated IFN-α–induced expression of ISGs (Fig. 6*A*). Furthermore, we assessed the expression profiles of FSK-regulated ISGs using 29 cross-genes (Fig. 6, *B* and *C*) and found that the addition of FSK regulated the differential expression of these ISGs (Fig. S6*A*). In addition, we derived a score using a 29-gene signature (Z-score and ISGs score; Fig. 6*D* and Fig. S6*B*) to quantify the involvement of the PKA-regulated IFN pathway in tumorigenesis. We identified changes in genetic signatures among the control, IFN-α, and FSK plus IFN-α groups as readings of IFN activity signaling. This analysis revealed the addition of FSK decreased the expression of ISGs induced by IFN-α. To investigate the PKA-mediated transcriptional regulatory activity of ISGs, a list of transcription factors (Fig. S6*C*) was obtained from an interferome database. The addition of FSK regulated the expression of these key transcription factors (Fig. 6*E*), thereby regulating related signaling pathways such as JAK/STAT and NF-κB. We identified 10 cross-linked SHP2 signature genes by differential gene expression analysis of FSK plus IFN-α and IFN-α groups (Fig. 6*F*) and found that the addition of FSK regulated the differential expression of SHP2 signature-related genes (Fig. 6*G*). These results suggest that PKA activation by FSK regulates JAK/STAT, SHP2, and NF-κB signaling.

### PGE_2_ suppresses activation of JAK/STAT through PKA/SHP2 signaling

**Figure 7:**
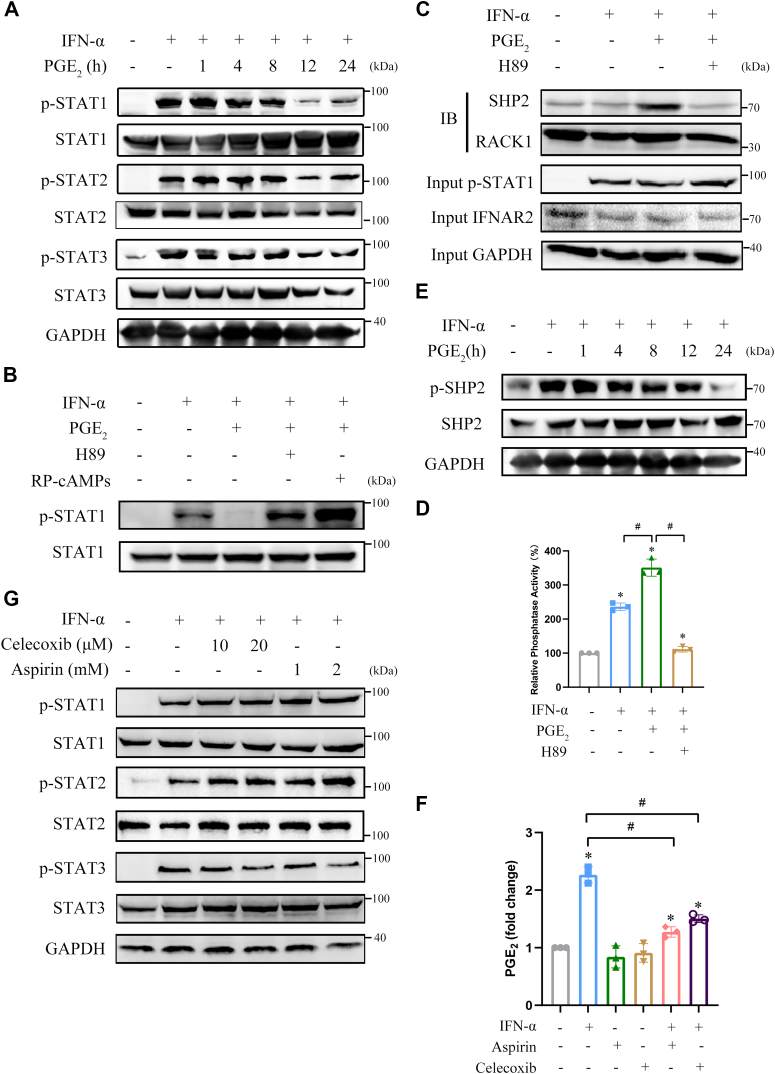
**PGE**_**2**_**suppresses activation of JAK/STAT through PKA/SHP2 signaling.***A*, Huh-7 cells were pretreated with DMSO or 10 μM PGE_2_ for the indicated time and then treated with IFN-α for 30 min. Cell lysates were immunoblotted using antibodies against phospho-STAT1 (Tyr701), phospho-STAT2 (Tyr690), phospho-STAT3 (Tyr705), STAT1, STAT2, and STAT3. *B*, HEK293A cells were pretreated with H89 or RP-cAMPs for 1 h and then treated with PEG_2_ for 10 min before treatment with IFN-α for 30 min. Cell lysates were immunoblotted using antibodies against phospho-STAT1 (Tyr701) and STAT1. *C* and *D*, HEK293A cell lysates were immunoprecipitated with the IFNAR2 antibody, and the co-immunoprecipitation products were divided into two parts. One sample was immunoblotted using SHP2 and RACK1 antibodies. In addition, 5% of the cell lysates were immunoblotted using antibodies against phospho-STAT1 (Tyr701) and IFNAR2. GAPDH staining was used as a loading control (*C*). The other sample was used to detect the phosphatase activity of SHP2 (*D*). ∗*p* < 0.05 *versus* control group, #*p* < 0.05 *versus* IFN-α + PGE_2_ treatment group (unpaired two-tailed Student’s *t* test). *E*, Huh-7 cells were pretreated with DMSO or 10 μM PGE_2_ for the indicated time and then treated with IFN-α for 30 min. Cell lysates were immunoblotted using antibodies against phospho-SHP2 (Tyr690) and SHP2 GAPDH was used as a loading control. *F*, HEK293A cells were pretreated with aspirin or celecoxib for 1 h before treatment with IFN-α for 30 min. Cell lysates were used to detect fold changes in PGE_2_ levels. ∗*p* < 0.05 *versus* control group, #*p* < 0.05 *versus* IFN-α treatment group (unpaired two-tailed Student’s *t* test). *G*, HEK293A cells were pretreated with aspirin or celecoxib for 1 h before treatment with IFN-α for 30 min. Cell lysates were immunoblotted using antibodies against phospho-STAT1 (Tyr701), STAT1, phospho-STAT2 (Tyr690), STAT2, phospho-STAT3 (Tyr705) and STAT3, DMSO, 0.1%; IFN-α, 5000 U/ml; H89, 10 μM; RP-cAMPs, 50 μM; PGE_2_, 10 μM. All experiments were conducted with three independent replicates and the results of representative data are shown. The data are presented as the mean ± SD from three independent experiments.

PGE_2_ is an abundant and potent tumor-promoting factor that exerts its physiological functions through cAMP/PKA signaling (18). Thus, we evaluated whether PGE_2_ exerts its tumor-promoting effects by modulating type I IFNs-induced signaling in a manner similar to that of FSK. PGE_2_ treatment significantly inhibited IFN-α–induced phosphorylation of STAT1, STAT2, and STAT3 in Huh-7 and HCCLM3 cells in a time-dependent manner (Fig. 7*A* and Fig. S7*A*). It also potently inhibited IFN-α–induced phosphorylation of STAT1 and mRNA expression of *PKR* and *2′,5′-OAS* in HEK293A cells (Fig. S7*B* and Fig. S1, *J* and *K*). Treatment with H89 or RP-cAMPs obviously reversed the suppression of PGE_2_ on STAT1 phosphorylation (Fig. 7*B*) and the mRNA expression of *PKR* and *2′,5′-OAS* (Fig. S1, *J* and *K*). Furthermore, PGE_2_ promoted the binding of SHP2 to IFNAR2, which was suppressed by H89 treatment (Fig. 7*C* and Fig. S7*C*). PGE_2_ also enhanced the phosphatase activity of SHP2 bound to IFNAR2, which was abrogated by H89 treatment (Fig. 7*D* and Fig. S7*D*). PGE_2_ also significantly inhibited IFN-α–induced phosphorylation of SHP2 at Tyr690 (Fig. 7*E*). The present results indicate that PGE_2_ suppresses IFN-α–mediated JAK/STAT pathway signaling *via* PKA-activated SHP2, similar to FSK. We then evaluated whether IFN-α induces COX2 expression to alter PGE_2_ production. As expected, IFN-α significantly stimulated the expression of COX2 (Fig. S7*E*), which was consistent with previous findings (19). The expression of COX2 is tightly regulated by a series of transcription factors including STAT3 and NF-κB (20), so we examined whether STAT3 and NF-κB participate in the regulation of IFN-α on COX2 expression. STAT3 inhibitor S3I-201 and IκB inhibitor BMS-345541 attenuated IFN-α–mediated COX2 protein induction, respectively (Fig. S7*E*). Similar results were obtained with STAT3 knockdown using siRNA (Fig. S7*F*). These findings suggest that IFN-α induces COX2 expression and PGE_2_ production by activating STAT3 and NF-κB. We then examined whether co-treatment with COX2 inhibitors is a promising strategy to enhance IFN-α–mediated signaling and action. Both aspirin and celecoxib, two well-known COX2 inhibitors, markedly repressed IFN-α–induced PGE_2_ production in Huh-7 cells (Fig. 7*F*). Moreover, IFN-α–induced tyrosine phosphorylation of STAT1 and STAT2 was further enhanced by celecoxib and aspirin treatment, respectively, whereas a reduction in the phosphorylation of STAT3 was observed in celecoxib- or aspirin-treated Huh-7 cells (Fig. 7*G*). These results suggest that PGE_2_ suppresses the activation of JAK/STAT induced by type I IFNs through PKA/SHP2 signaling, which can be counteracted by COX2 inhibitors.

### Aspirin promotes the antiproliferative effect of IFN-**α***in vivo*

**Figure 8:**
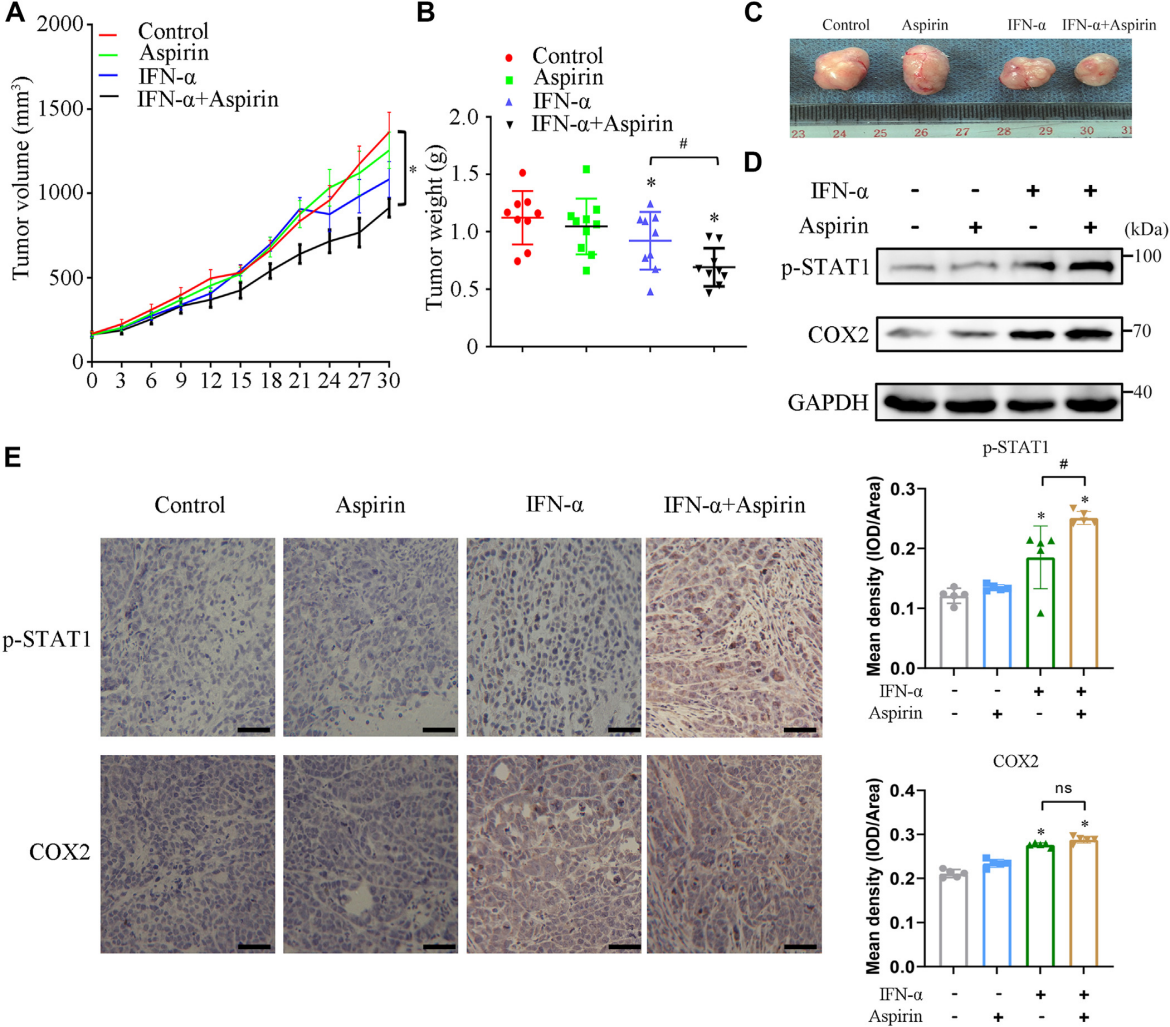
**Aspirin promotes the antiproliferative effect of IFN-α *in vivo*.***A–C*, xenograft tumor (Huh-7 cells) mice were treated with aspirin or IFN-α plus aspirin for 30 days and the tumor growth, tumor weight, and representative images are shown (n = 9, each group). ∗*p* < 0.05 *versus* control group, #*p* < 0.05 *versus* IFN-α group-treated group (unpaired two-tailed Student’s *t* test). *D* and *E*, the phosphorylation of STAT1 and expression of COX2 in tumor tissues were detected by Western blotting and immunohistochemistry (Scale bar represents 20 μm), respectively. ∗*p* < 0.05 *versus* control group, #*p* < 0.05 *versus* IFN-α treatment group (unpaired two-tailed Student’s *t* test). ns, nonsignificant. IFN-α, 1 × 10^5^ U/kg; Aspirin, 15 mg/kg. All experiments were conducted with three independent replicates and the results of representative data are shown. The data are presented as the mean ± SD from three independent experiments.

Next, we examined whether COX2 inhibitor aspirin could enhance the anticancer activity of IFN-α *in vivo*. The nude mice bearing Huh-7 HCC cells was established and then treated with IFN-α, aspirin, or IFN-α plus aspirin, respectively. As shown in Fig. 8, *A–C*, treatment with IFN-α alone caused a moderate suppression of tumor volume and tumor weight, with reduction of about 18%. Notably, treatment with IFN-α plus aspirin resulted in a synergistic inhibition on tumor growth, with reduction of about 38%. Furthermore, the effects of aspirin on IFN-α–induced activation of STAT1 and induction of COX2 were evaluated in tumor tissues using Western blot assays. As shown in Fig. 8*D*, treatment of IFN-α alone upregulated the tyrosine phosphorylation of STAT1 in tumor tissues compared with the control group and aspirin group and the combination treatment of IFN-α plus aspirin further enhanced the phosphorylation of STAT1 compared with that of IFN-α treatment alone. However, IFN-α–induced elevated expression of COX2 was not attenuated by aspirin treatment (Fig. 8*D*). Similar results for STAT1 phosphorylation and COX2 expression were obtained by immunohistochemistry (Fig. 8*E*).

To further clarify the role of PKA and SHP2 in the anti-HCC effect of IFN-α *in vivo*, the nude mice bearing Huh-7 HCC cells were established and then treated with IFN-α, SHP2 inhibitor SHP099, PKA inhibitor H89, IFN-α plus SHP099, IFN-α plus H89, respectively. As shown in Fig. S8, *A–C*, treatment with IFN-α, SHP099, or H89 alone caused an effective inhibition of tumor weight, with reduction of about 48%, 40.8%, and 15.2%, respectively, whereas treatment with IFN-α in combination with SHP099 and H89 synergistically inhibited tumor growth, reducing it by approximately 82.6% and 77.2%, respectively. In addition, compared to treatment with IFN-α alone, the combination of IFN-α with SHP099 and H89 further enhanced the phosphorylation of STAT1 in the nucleus and suppressed the expression of COX2 (Fig. S8*F*). These results suggested that inhibition of PKA and SHP2 enhanced anticancer effects of IFN-α, consistent with their *in vitro* observation *in vivo*.

### Expressions of STAT1/3-SHP2-COX2 axis are correlated with the human HCC development

To determine whether the expression of these proteins was aberrant in human HCC, we used liver tissue microarrays to analyze a cohort of 90 patients with HCC. The level of p-STAT3, especially nuclear p-STAT3, was elevated in HCC tumors compared to that in adjacent normal liver tissues (Fig. S9*A*). Statistically, COX2 and p-STAT1 expression was significantly lower in HCC tumor tissues than in adjacent normal liver tissues, and nuclear pTyr-STAT3 expression was significantly higher in HCC tumor tissues than in adjacent normal liver tissues. However, no variation in SHP2 expression was observed (Fig. S9, *A* and *B*). Correlation analysis was also performed and the expression of COX2 and p-STAT3 was found to be positively correlated with the development of HCC (Fig. S9*C*). Furthermore, lower expression of COX2 was observed in HCC tumor tissues, which correlated with a worse outcome when compared with adjacent normal liver tissues (Fig. S9*D*). In addition, patients with HCC who have higher p-STAT3 levels exhibited poor clinical outcomes, and the median survival time of the high p-STAT3 group *versus* and low p-STAT3 groups were 28 and 45 months, respectively (Fig. S9*E*). We also analyzed the correlation between p-STAT1 and SHP2 expression and the survival of patients with HCC. However, no significant correlation was found between the expression of these two proteins and survival of patients with HCC (Fig. S9, *F* and *G*). These findings indicate that high expression of COX2 and p-STAT3 may be correlated with HCC development and poor prognosis in patients with HCC.

## Discussion

During clinical use of IFN-α, patients often develop resistance to it, but the mechanisms of resistance are not yet fully understood. Several negative regulatory mechanisms, such as IFNAR degradation and the action of suppressors of cytokine signaling proteins, are likely responsible for IFN-α resistance (33). Hence, understanding these negative regulation mechanisms of IFN-α–induced JAK/STAT signaling may contribute to improving IFN-α resistance and enhancing its clinical efficacy. Previously, we reported that PDE/cAMP/PKA signaling, which is integrated by RACK1 into IFNAR2, negatively regulates IFN signaling through SHP2 (17). Thus, inhibiting this negative pathway may provide a new strategy to boost the anti-cancer efficacy of IFN-α/β. However, the precise mechanism by which PKA regulates IFN signaling remains unclear. In this study, we found that activation of PKA promoted the binding of SHP2 to IFNAR2 and STAT1 and increased the local phosphatase activity of SHP2 on IFNAR2, suggesting that the negative role of PKA in the JAK/STAT pathway is mediated by SHP2 activation. Phosphorylation is critical for the activity of SHP2. For example, exogenous stimulations by cytokines such as EGF, FGF2, PDGF, and hGF upregulate SHP2 activity by increasing Tyr542 phosphorylation, promoting cell growth (34). In the present study, we found that IFN-α also induced Tyr542 phosphorylation of SHP2 in HCC cells. However, this effect can be suppressed by FSK treatment, resulting in increased SHP2 activity. This suggests that the crosstalk between the cAMP/PKA and JAK/STAT pathways could have a unique regulatory effect on Tyr542 phosphorylation of SHP2, a key phosphorylation site indicative of SHP2 activity. We found that PKA increased the activity of SHP2 by phosphorylating serine234 in response to IFN-α stimulation. Thus, PKA-mediated Ser234 phosphorylation might induce structural changes in the PTP domain of SHP2 to prevent IFN-α–induced Tyr542 phosphorylation, which needs to be further investigated. Interestingly, PKA-mediated phosphorylation of SHP2 at Thr73/Ser189 inhibits tyrosine-phosphorylated ligand binding and PTP activity following β-adrenergic stimulation (20). Therefore, PKA-mediated phosphorylation of SHP2 at different residues may exhibit distinct PTPase activity in diverse cellular contexts. A recent study reported that the phosphorylation of SHP2 at Tyr62 stabilizes SHP2 in an open conformation and mediates resistance to certain SHP2 inhibitors in acute myeloid leukemia (35). Therefore, considering the important role of the cAMP/PKA pathway in the mediation of multiple cytokines or hormones, further investigation is warranted to determine whether phosphorylation of SHP2 at Ser234 mediates resistance to clinically investigated SHP2 inhibitors.

Adaptors such as AKAPs are responsible for the specific subcellular localization of PKA by forming signaling complexes with phosphatases or kinases, thereby dynamically regulating compartmentalized cAMP signaling (36). The anchoring protein RACK1 was found to interact with PDE4D5 during cAMP hydrolysis and SHP2 activation by PKC (37, 38). Additionally, we found that RACK1 physically interacts with PKA *via* the WD-3/4 domain, which further highlights the strong association between RACK1 and cAMP/PKA pathway. We previously revealed that RACK1 is involved in the modulation of IFN-α signaling by orchestrating PKA and SHP2 at IFNAR2 (17). However, the mechanism by which RACK1 regulates SHP2 remained unknown. Activated PKA promoted the disassociation of RACK1 from SHP2 and enhanced the local PPTase activity of SHP2 at RACK1. Further, activated PKA enhanced the association of SHP2 with STAT1 and IFNAR2. Thus, dissociated SHP2 from RACK1 may directly anchor to the local sites of IFNAR2 and STAT1, thereby attenuating type I IFN-induced JAK/STAT signaling. However, further investigation is required to determine whether PKA phosphorylation of SHP2 at Ser234 facilitates the disassociation of SHP2 from RACK1. Although RACK1 recruits multiple proteins to the insulin-like growth factor receptor I, including Shc, insulin receptor substrate-1/2, SHP2, and STAT3 (39), it remains unclear how RACK1 orchestrates the crosstalk between cAMP signaling and the JAK/STAT pathway. The findings of the present study revealed that PDE4D also participates in the negative regulation of IFN-stimulated JAK/STAT signaling, revealing a new function of PDE4D in the regulation of the JAK/STAT pathway in HCC. IFNAR2 region RACK1 physically interacted with PKA and PDE4D, which form a signal complex to modulate the compartmentalized cAMP signaling by PKA-mediated SHP2 dissociation and activity. This observation is consistent with our previous observation in bladder cancer cells, where IFN-α enhances the PDE4D activity to reduce the cAMP level through a dynamic interaction between IFNAR2 and PDE4D (40). Considering that cAMP is widely stimulated by multiple cytokines, the PKA/PDE4D/SHP2/RACK1 signaling complex formed in IFNAR2 may provide a new regulatory layer to spatiotemporally modulate the duration and activation of type I IFNs-induced JAK/STAT pathway signaling. The suppressive function of Treg cells is eliminated through a pathway involving MEK/ERK-mediated PDE4 activation and the consequent depletion of cAMP (41). Additionally, mumps virus protein V blocks IFN signal transduction by reducing STAT1 production as it has a higher affinity than RACK1-STAT1. This impairs IFN signaling by disrupting the complex composed of STAT1, RACK1, and the IFN receptor (42). Thus, hijacking the RACK1 complex in the IFN receptor is a natural strategy that was evolved in viruses to counteract the antiviral effects of IFNs. RACK1 is highly expressed in the normal liver and frequently upregulated in HCC, and its expression correlated well with poor clinical progression of HCC (43). RACK1 can exert partner-specific functions and participate in diverse biological events; however, it remains unclear how RACK1 can distinguish messages from different partners. In this study, we found that the RACK1 complex that localizes to IFNAR2 can sense cAMP signaling stimulated by other cytokines to modulate IFN signaling, which plays a key role in mediating crosstalk between IFNs and other cytokines.

It is a recognized hallmark of cancer that nonresolving inflammation substantially contributes to the development and progression of HCC, leading to the increased production of PGE_2_ and promoting the proliferation and migration of HCC cells through multiple pathways majorly related with PKA (23, 44). Enhanced COX2 expression in hepatocytes was sufficient to induce spontaneous HCC formation in mice (26). It was reported that the combined regimen with celecoxib and IFN-α reduced the growth of xenotransplanted HCCs by increasing the expression of TRAIL and its receptors (45). Here, we further found that type I IFNs induced the expression of COX2 expression and PGE_2_ production by STAT3/NF-κB pathway, and PGE_2_ activates SHP2 with the similar mechanism as FSK, an agonist of adenylyl cyclase. The combination treatment of IFN-α and COX2 inhibitor aspirin suppressed the proliferation of HCC cells more efficiently than alone treatment *in vivo*, consistent with the observation that combination of COX2-specific inhibitor NS-398 with IFN-β reduced the growth of xenotransplanted HCCs in nude mice (30). IFN-α is widely used for the treatment of HBV or HCV; however, its clinical efficacy is severely limited by associated side effects such as hematological toxicities and drug tolerance. PGE_2_ levels are significantly increased in the tumor microenvironment, and PGE_2_ has emerged as an important player in immune cell activation and migration (22). Our observation that type I IFNs induce COX2 expression and PGE_2_ production may provide new insights into the understanding of drug tolerance after long-term IFN-α treatment. In addition to their direct antiproliferative effect on cancer cells, type I IFNs are generally considered beneficial for antitumor immunity by promoting T cell activation. Surprisingly, sustained type I IFN signaling is associated with resistance to immune checkpoint blockade (ICB) therapy in patients with melanoma (46). Recently, chronic type I interferon signaling was found to promote lipid peroxidation–driven terminal CD8^+^ T-cell exhaustion and curtail anti-PD-1 efficacy (47). Therefore, increased PGE_2_ production induced by chronic type I IFNs treatment may provide an immunosuppressive microenvironment. Currently, many type I IFN inducers, such as STING or TLR agonists, are used alone or in combination with ICBs to treat tumors in preclinical models and clinical trials. However, their antitumor effects are unsatisfactory under certain circumstances, possibly due to the side effects caused by type I IFNs (3). Therefore, PGE_2_ or COX2 expression should be considered in a specific tumor background to determine the therapeutic schedule and whether COX2 inhibitors can be used to enhance the cancer immunotherapeutic effect of type I IFNs alone or in combination with ICBs needs to be further investigated.

In this study, using clinical HCC samples, we found that higher expression of COX2 and p-STAT3 may be correlated with HCC development and poor prognosis in patients with HCC. This is consistent with the observation that STAT3 is constitutively active in up to 60% of the HCC cases (48). The expression of p-STAT1 was found to be lower in HCC tumor tissues than in adjacent normal liver tissues, consistent with the observation that the suppression of STAT1 activity was correlated with HCC progression and prognosis in a set of HCC patient samples (49). However, we found that the expression of COX2 was higher in adjacent normal liver tissues than in cancerous tissues, which was inconsistent with the observation that higher COX2 expression was identified in HCC tumors than in normal liver tissues (50). This discrepancy is possibly caused by the differentiated HCC clinical samples used because it was also found that less-differentiated HCC tissues expressed less COX2 than hepatocytes of adjacent nontumorous livers (27). Although elevated COX2 expression is typically associated with inflammation and tumor progression, its complex role within different tumor microenvironments suggests it may also contribute to immune responses in certain cancer types, potentially leading to improved patient prognosis. Further research is needed to elucidate the mechanisms by which COX expression influences prognosis and to explore its potential as a biomarker and therapeutic target in HCC or other cancer subtypes. Therefore, higher PGE_2_ levels can form in the tumor microenvironment of well-differentiated and less-differentiated HCCs and directly interfere with the function of type I IFNs. However, the role and correlation of SHP2 expression with HCC prognosis and development remains controversial (51, 52). In this study, we found that SHP2 was not differentially expressed in HCC tumor tissues and adjacent normal tissues and was correlated with the prognosis and development of patients with HCC, indicating that changes in the activity of SHP2 protein rather than changes in its protein expression should be examined to monitor its oncogenic function. SHP2 was found to promote breast cancer progression and sustain tumor-initiating cells *via* the activation of key transcription factors and a positive feedback signaling loop. However, consistent differences in SHP2 expression between normal and neoplastic breast cancer tissues were not observed, and SHP2 expression did not significantly correlate with any tumor histotype or clinicopathological parameters (53). Therefore, SHP2 activation—and not SHP2 expression—may play a crucial role not only in breast tumorigenesis but also in HCC tumorigenesis.

**Figure 9:**
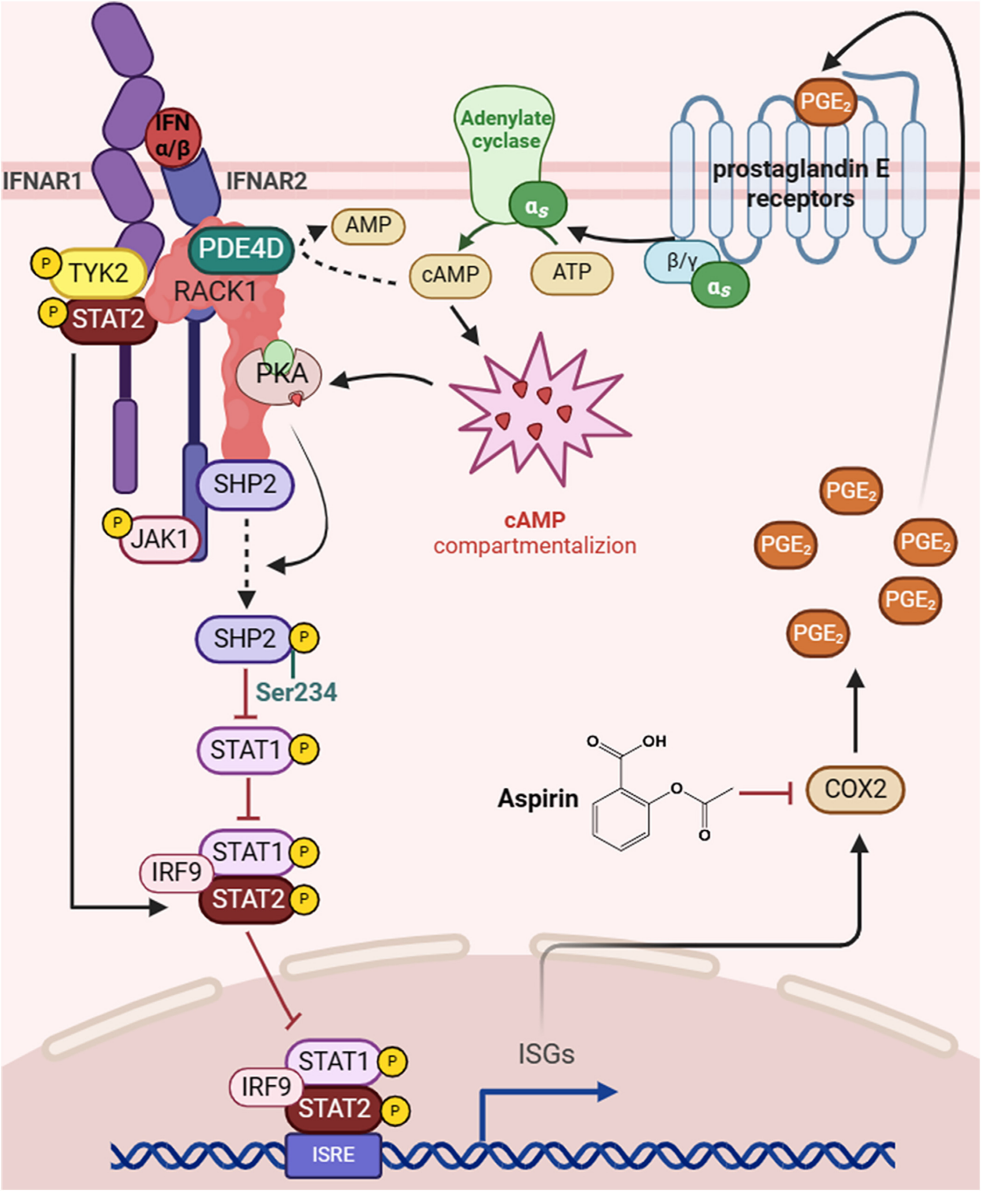
**Schematic depiction of the PKA/SHP2/STAT1 pathway.** Elevated intracellular cAMP levels and activation of PKA can enhance PPTase activity of SHP2 and ultimately attenuate type I IFNs-induced activation of JAK/STAT signaling. In addition, type I IFN can upregulate the expression of COX2 and production of PGE_2_, thus forming a negative feedback loop by activating PKA/SHP2 axis to attenuate the JAK/STAT pathway signaling.

In summary, we have shown that elevated intracellular cAMP levels and activation of PKA may enhance the PPTase activity of SHP2, ultimately attenuating type I IFNs-induced activation of JAK/STAT signaling and the anti-proliferative action in HCC cells. Furthermore, type I IFNs can upregulate the expression of COX2 and production of PGE_2_ through activating the STAT3-NF-κB pathway, thus forming a negative feedback loop by activating PKA–SHP2 axis to attenuate the JAK/STAT pathway signaling (Fig. 9). These findings revealed a fundamental PKA/SHP2-dependent negative feedback loop acting on JAK/STAT signaling, and inhibition of this signaling by the selective COX2 inhibitors may enhance the clinical efficacy of type I IFNs in treating HCC.

## Experimental procedures

### Cell culture

Huh-7, HCCLM3 HCC cells, and HEK293A cells were obtained from the Cell Bank of the Chinese Academy of Sciences. Cells were cultured in Dulbecco’s modified Eagle’s medium containing 10% fetal bovine serum and 1% penicillin/streptomycin (Hyclone) at 37 °C in humidified air with 5% CO_2_. These cells were authenticated by Genetic Testing Biotechnology using short tandem repeat profiling and examined for *mycoplasma* contamination.

### Reagents and antibodies

FSK, H89, RP-cAMPs, KT-5720, celecoxib, and aspirin were purchased from Selleck. IFN-α (recombinant human IFN-α2a) was purchased from GenScript. Phospho-Tyr-701-STAT1 (#11044, 1: 1000 for WB; 1: 50 for IHC), Phospho-Tyr-705-STAT3 (#11045, 1: 1000 for WB) antibodies were purchased from Signaling Antibodies, whereas STAT1 (#10144-2-AP, 1: 1000 for WB; 1 μg/mg lysate for IP), STAT2 (#16674-1-AP, 1: 1000 for WB), and STAT3 (#10253-2-AP, 1: 1000 for WB), SHP2 (#20145-1-AP, 1: 1000 for WB; 1 μg/mg lysate for IP; 1: 200 for IF), IFNAR2 (#10522-1-AP, 1: 1000 for WB; 1 μg/mg lysate for IP; 1: 200 for IF), and COX2 (#27308-1-AP, 1: 1000 for WB) antibodies were purchased from Proteintech. RACK1 (#5432, 1: 1000 for WB) antibody was purchased from Cell Signaling Technology;, pan Phospho-Serine/Threonine Rabbit Polyclonal Antibody (#AF5725, 1: 1000 for WB) was purchased from Beyotime.

### Real-time quantitative reverse transcription-PCR

Total cellular RNA was extracted using the TRIzol reagent (Invitrogen) according to the manufacturer's instructions. Total RNA was reverse transcribed using the M-MLV enzyme with an oligo dT18 primer (Promega). Equal amounts of cDNA were amplified using real-time quantitative PCR with specific primers for *PKR*, 2′,5′-OAS1, and *GAPDH* (*PKR*: 5′-GTT TGC TTC TCT GGC GGT CTT-3′ and 5′-GCC ATT TCT TCT TCC CGT ATC C-3′; *2′,5′-OAS1*:5′-AGG TGG TAA AGG GTG GCT CC-3′ and 5′-ACA ACC AGG TCA GCG TCA GAT-3′; *GAPDH*: 5′-TGC ACC ACC AAC TGC TTA GC-3′ and 5′-GGC ATG GAC TGT GGT CAT GAG-3′). The PCR products were detected using fluorescent Maxima SYBR Green (Thermo Fisher Scientific). The quantities of *PKR* and *2′,5′-OAS1* mRNA were normalized to those of *GAPDH* mRNA in the same samples.

### RNA interference

Synthetic siRNA probes against SHP2 and STAT3 were purchased from RIBOBIO. HEK293 A and Huh-7 cells were seeded in 6-well plates. After incubation for 24 h, the cells were transfected with 50 nM of a specific siRNA or siRNAcon (control) for 72 h using Lipofectamine2000 (Thermo Fisher Scientific) according to the manufacturer’s specifications.

### Protein purification and GST pull-down

GST-fusion proteins, including GST-IFNAR2, GST-PKA, GST-SHP2, GST-RACK1, and their mutants were expressed in *Escherichia coli* BL21 cells and purified using glutathione-coated beads (Amersham Pharmacia) according to the manufacturer’s instructions. For the GST pulldown assay, the cells were lysed in TNE buffer (50 mM Tris–HCl, pH 7.4, 100 mM NaCl, 0.1 mM EDTA, 1% protease inhibitor). The cell lysates were centrifuged at 14,000×*g* for 5 min at 4 °C. The supernatants were incubated with GST-fusion proteins and GST-beads overnight at 4 °C. The beads were washed with excess wash buffer, resuspended in SDS loading buffer, and boiled for 5 min. Supernatants were loaded onto SDS-PAGE gels, followed by immunoblotting.

### Western blot analysis and Co-IP

After specific treatments, cells were lysed with RIPA buffer containing 1% protease inhibitor (Sigma). Cell lysates were subjected to SDS-PAGE. Proteins were then transferred onto nitrocellulose membranes (Millipore). The membranes were blocked with tris-buffered saline containing 0.1% Tween 20 (TBST) containing 5% bovine serum albumin and incubated with primary antibodies (1:1000) overnight at 4 °C. After three washes with TBST, the membranes were incubated with horseradish peroxidase–conjugated secondary antibodies (1:5000; Proteintech) for 1 h at room temperature and washed with TBST three times. Signals were detected using chemiluminescence (Beyotime) and quantified using Quantity One (Bio-Rad).

For the Co-IP assay, cells were lysed with NP-40 buffer (1% NP-40, 50 mM Tris–HCl [pH 7.5], 150 mM NaCl, 1 mM EDTA, and 1 mM MgCl_2_, 1% protease inhibitor). The lysates were centrifuged at 14,000×*g* for 5 min at 4 °C. The supernatants (1 mg protein) were then incubated with 1 μg indicated antibodies followed by incubation with 20 μl protein A/G-agarose beads (Santa Cruz). The beads were washed thrice with pre-cooled NP-40 buffer and boiled in SDS loading buffer.

### Phosphorylation of SHP2 by PKA *in vitro*

Purified recombinant GST-SHP2 (0.5 μg) and 0.5 μg of point mutation of GST-SHP2-S234A or GST-SHP2-S365A were incubated with PKA (83 U/μl) in the presence of 1000 μCi^γ-32P^ ATP for 30 min at 37 °C in 30 μl reaction system and then mixed in 5 μl 5 × loading buffer, boiled for 5 min. The samples were then separated using SDS-PAGE, and phosphorylated SHP2 was detected by autoradiography and immunoblotting.

### Determination of SHP2 activity

GST-SHP2 (0.5 μg), PKA (83 U/μl), DIFMUP (10 μM), ATP (10 μM), and reaction buffer (50 mM Hepes, 150 mM NaCl, 1 mM EDTA, 2 mM DTT, pH = 7.0); or the products of GST pull-down or IP, DIFMUP (10 μM), ATP (10 μM), and reaction buffer were incubated for 30 min in 100 μl reaction buffer at 37 °C in the presence and absence of Na_3_VO_4_ (50 μM). Phosphatase activity of SHP2 was detected using a microplate reader (excitation wavelength, 355 nm; emission wavelength, 460 nm).

### Immunofluorescent assay

Briefly, cells (2 × 10^5^ cells/dish) cultured into glass-bottom culture dish overnight and then treated with H89 for 1 h and followed by incubation with FSK for 45 min prior to treatment of IFN-α for 30 min. The cells were washed with PBS, fixed with 4% PFA for 15 min, and permeabilized with 0.1% Triton X-100 for 15 min at room temperature. After blocking with 5% bovine serum albumin, cells were incubated with the mouse anti-IFNAR2, rabbit anti-PKA RII, or rabbit anti-SHP2 (1:200) overnight at 4 °C and followed by incubation with a Alexa fluor 488–labeled goat anti-mouse IgG and Alexa fluor 555–labeled donkey anti-rabbit IgG (1:1000, Thermo Fisher Scientific) for 1 h at room temperature. Nuclei were stained with DAPI (0.5 μg/ml, sigma) for 5 min before observation. Fluorescence was analyzed using a Leica TCS SP8 confocal microscope (Jena).

### RNA-seq and data analysis

Total RNA was extracted from the Huh-7 cells, and mRNA was purified from the total RNA using poly T oligo-attached magnetic beads. An RNA library was constructed using the AMPure XP system (Beckman Coulter) and library preparations were sequenced on an Illumina Novaseq platform (Shanghai Applied Protein Technology Co., Ltd). Differential expression analysis of ctrl, IFN α-, and FSK + IFN α-treated group was performed using the DESeq2 R package (1.20.0). Genes with a *p*-value < 0.05, detected by DESeq2, were considered differentially expressed. Differentially expressed genes were detected in the Kyoto Encyclopedia of Genes and Genomes pathway using the clusterProfiler R package. GSEA was performed using the GSEA analysis tool (http://www.broadinstitute.org/gsea/index.jsp) for functional gene annotation. ISGs scores were calculated by the normalized log2 (N_gene expression data_ + 1) from ISGs signatures.

### Animal study

All mouse experiments were conducted according to the animal protocols approved by the Institutional Animal Care and Use Committee. For tumor xenograft studies, Huh-7 cells (5 × 10^6^ cells in 200 μl) were injected subcutaneously into the flank region of female 6-week-old nude mice (Beijing Vital River Laboratory Animal Technology Co., Ltd). After tumor xenografts reached approximately 100 mm^3^ (0.5 × length × width^2^), the mice were treated with the indicated drugs for 4 weeks. Finally, the animals were sacrificed under carbon dioxide anesthesia, and the tumors were resected and weighed. Tumors were then cut into two parts: one was fixed with 4% PFA and used for the immunohistochemistry assay, and the other was used for the Western blot assay.

### Enzyme-linked immunosorbent assay

The level of PGE_2_ was measured as follows: briefly, cells were treated with indicated agents, the cell culture mediums were then harvested and centrifugated at 1000 g at 4 °C for 5 min. Furthermore, tumors were homogenated and centrifugated at 12,000 g at 4 °C for 5 min. The supernatants were used to determine PGE_2_ levels *via* ELISA according to the manufacturer's protocol.

### Tissue microarray

Immunohistochemistry of the human HCC tissue array (Shanghai Outdo Biotech Co. Ltd) was performed using antibodies directed against pTyr-701 STAT1, pTyr-705 STAT3, SHP2, and COX2. This tissue microarray included 90 cases of HCC tumors (one core/case) and matched normal adjacent tissue (one core/case). Information regarding clinical stage, survival information, smoking history, drinking history, diabetes history, hepatitis B information, and family medical history were obtained from the Outdo website (http://www.superchip.com.cn/index.html).

### Statistical analysis

All experiments were repeated at least three times, and representative results are presented. Data are expressed as mean values ± SD. Statistical analyses were performed using GraphPad Prism software (version 5.0; GraphPad) and statistical significance among experimental groups was evaluated using unpaired two-tailed Student’s *t* test. Differences were considered statistically significant at *p* < 0.05.

## Data availability

All datasets used and/or analyzed during the current study are available from the corresponding author on reasonable request.

## Supporting information

This article contains supporting information.

## Conflict of interest

The authors declare that they have no conflicts of interest with the contents of this article.

## Supporting information

Figurs S1-S9
