# Supplementary material for: Protein kinase a suppresses antiproliferative effect of interferon-α in hepatocellular carcinoma by activation of protein tyrosine phosphatase SHP2
Source: J Biol Chem. 2025 Jan 16;301(2):108195. doi: 10.1016/j.jbc.2025.108195 (PMC11849638; doi:10.1016/j.jbc.2025.108195)
Supplement: Figurs S1-S9 [file mmc1.pdf]

Protein Kinase A Suppresses Anti-proliferative Effect of Interferon-α in Hepatocellular Carcinoma by Activation of Protein Tyrosine Phosphatase SHP2

Yuwen Sheng[1,†], Yuan Lin[1,†], Zhe Qiang[1,2 †], Xiaofei Shen[1,3], Yujiao He[1,4], Lingyu Li[1,5], Sheng Li[1], Guolin Zhang[1], Fei Wang[1,*]

[1] Center for Natural Products Research, Chengdu Institute of Biology, Chinese Academy of Sciences, Chengdu, China

[2] Chongqing Academy of Chinese Materia Medica, Chongqing, China

[3] Hospital of Chengdu University of Traditional Chinese Medicine, Chengdu, China

[4] Anti-infective Agent Creation Engineering Research Centre of Sichuan Province, Sichuan Industrial Institute of Antibiotics, School of pharmacy, Chengdu University, Chengdu, China

[5] University of Chinese Academy of Sciences, Beijing, China

[†] These authors contributed equally to this work.

[*] **Correspondence**

Fei Wang, Center for Natural Products Research, Chengdu Institute of Biology, Chinese Academy of Sciences, Chengdu, China

Phone/Fax: +86 28 82890651, E-mail: wangfei@cib.ac.cn.

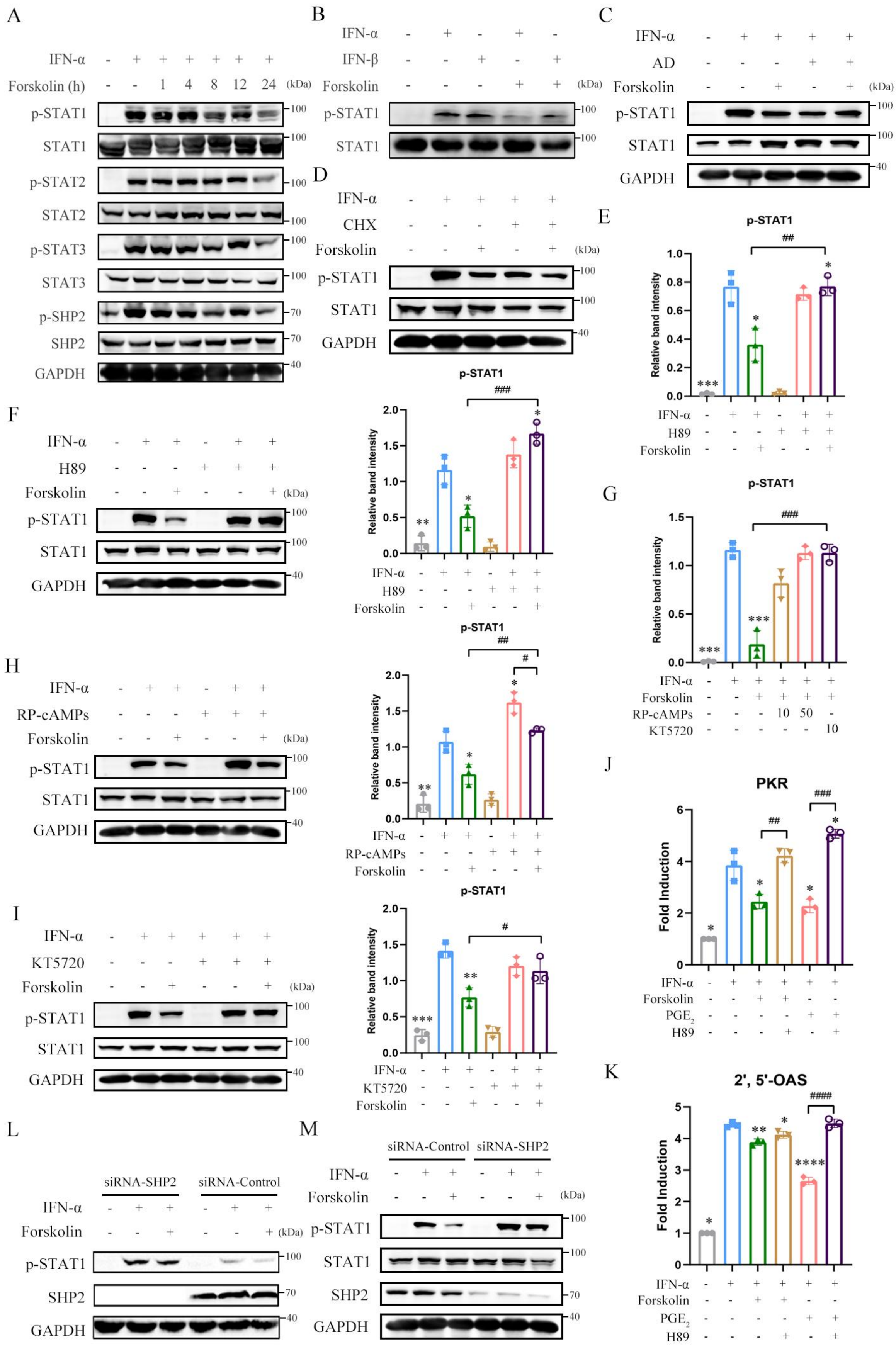
A
IFN-α
Forskolin (h)
1 4 8 12 24
(kDa)
p-STAT1
STAT1
p-STAT2
STAT2
p-STAT3
STAT3
p-SHP2
SHP2
GAPDH
100
70
40
B
IFN-α
IFN-β
Forskolin
C
IFN-α
AD
Forskolin
D
IFN-α
CHX
Forskolin
E
p-STAT1
##
Relative band intensity
IFN-α
H89
Forskolin
F
IFN-α
H89
Forskolin
p-STAT1
###
G
p-STAT1
###
IFN-α
Forskolin
RP-cAMPs
KT5720
10 50
10
H
IFN-α
RP-cAMPs
Forskolin
p-STAT1
##
#
I
IFN-α
KT5720
Forskolin
p-STAT1
#
J
PKR
Fold Induction
##
###
IFN-α
Forskolin
PGE2
H89
K
2', 5'-OAS
####
Fold Induction
IFN-α
Forskolin
PGE2
H89
L
siRNA-SHP2
siRNA-Control
IFN-α
Forskolin
p-STAT1
SHP2
GAPDH
M
siRNA-Control
siRNA-SHP2
IFN-α
Forskolin
p-STAT1
STAT1
SHP2
GAPDH

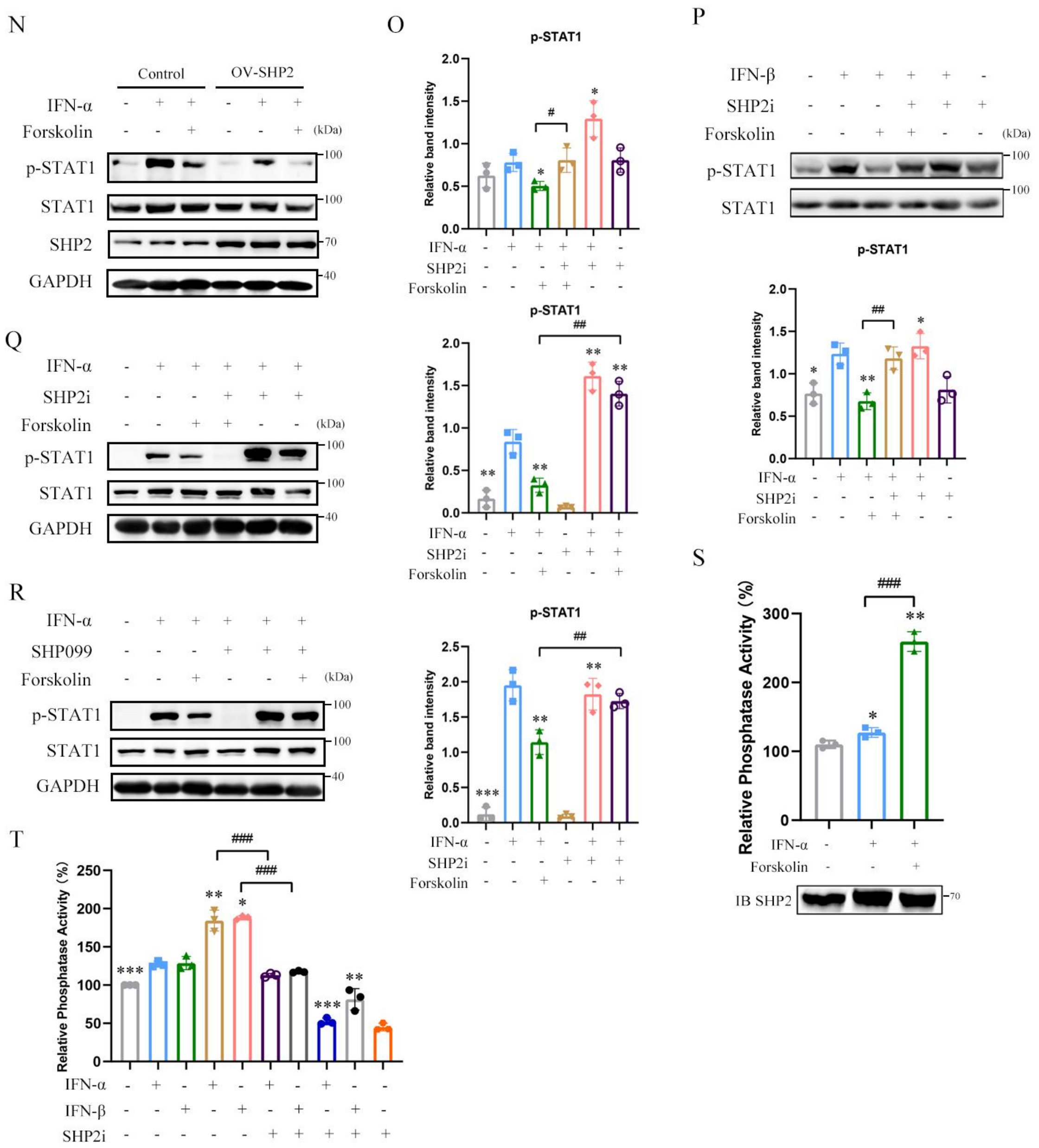

Fig. S1 PKA suppresses JAK/STAT pathway signaling via SHP2. *A,* HCCLM3 cells were pretreated with DMSO or 50 μM forskolin for the indicated time, and then treated with IFN-α for 30 min. Cell lysates were immunoblotted with antibodies against phospho-STAT1 (Tyr701), phospho-STAT2 (Tyr690), phospho-STAT3 (Tyr705), STAT1, STAT2, STAT3 and GAPDH antibodies. *B,* HEK293A cells were pretreated with DMSO or 50 μM forskolin for 45 min before treatment with IFN-α/β for 30 min. Cell lysates were immunoblotted using antibodies against phospho-STAT1 (Tyr701) and STAT1. *C, D,* Huh-7 cells were pretreated with DMSO, 50 μM

forskolin, 5 μM AD (*C*) or 5 nM CHX (*D*) for 4 h before treatment with IFN-α for 30 min. Cell lysates were immunoblotted using antibodies against phospho-STAT1 (Tyr701), STAT1 and GAPDH. *E, G,* Quantification of the p-STAT1 levels in the western blotting analysis (Fig. 1B) was performed, with protein levels normalized to STAT1 and expressed as relative values. *$p < 0.05$, ***$p < 0.001$ vs. IFN-α-treated group, ##$p < 0.01$, ###$p < 0.001$ vs. IFN-α + forskolin-treated group (unpaired two-tailed Student's *t*-test). *F, H, I, P-R,* Huh-7 cells were pretreated with DMSO, 10 μM H89 (*F*), 50 μM RP-cAMPs (*H*), 10 μM KT5720 (*I*), 200 μM SHP2 inhibitor (SHP2i) (*P, Q*) and 10 μM SHP099 (R) for 1 h and then with forskolin for 4 h before treatment with IFN-α/β for 30 min. Cell lysates were immunoblotted using antibodies against phospho-STAT1 (Tyr701), STAT1 and GAPDH. Quantification of the p-STAT1 levels in the western blotting analysis was performed, with protein levels normalized to STAT1 and expressed as relative values. *$p < 0.05$, **$p < 0.01$ and ***$p < 0.001$ vs. IFN-α-treated group, #$p < 0.05$, ##$p < 0.01$, and ###$p < 0.001$ vs. IFN-α + forskolin -treated group (unpaired two-tailed Student's *t*-test). *J, K,* After the pretreatment of PGE_2 (10 nM) or forskolin (50 μM) for 30 minutes and then PKA inhibitor (H89, 10 μM) for 45 min, Huh-7 cells were stimulated by IFN-α (2000 U/mL) for 20 h. The mRNA expression of *PKR* (*J*) and *2′,5′-OAS* (*K*) were measured by qRT-PCR. The results are presented as induction (n-fold) relative to basal levels in untreated cells. GADPH was used as the internal control. *$p< 0.05$, **$p< 0.01$ and ****p< 0.0001 vs. IFN-α-treated group, #$p < 0.05$, ##$p < 0.01$ and ###$p < 0.001$ vs. IFN-α + forskolin/PGE_2-treated group (unpaired two-tailed Student's *t*-test). *L,* HEK293A cells were transfected with siRNAcon (50 nM) or SHP2 siRNA002 (50 nM). After 72 h, the cells were pretreated with forskolin for 45 min and then incubated with IFN-α for 30 min. The cell lysates were immunoblotted using antibodies against phospho-STAT1 (Tyr701), STAT1, SHP2, and

GAPDH. *M, N,* Huh-7 cells were transfected with siRNAcon (50 nM) or SHP2 siRNA001 (50 nM) (*M*) and pCMV3-SHP2 plasmids (*N*). After 72 h, the cells were pretreated with forskolin for 4 h and then incubated with IFN-α for 30 min. The cell lysates were immunoblotted using antibodies against phospho-STAT1 (Tyr701), STAT1, SHP2, and GAPDH. *O,* Quantification of the p-STAT1 levels in the western blotting analysis (Fig. 1D) was performed, with protein levels normalized to STAT1 and expressed as relative values. *$p < 0.05$ vs. IFN-α-treated group, #$p < 0.05$ vs. IFN-α + forskolin-treated group (unpaired two-tailed Student's *t*-test). *S,* HEK293A cells were pretreated with DMSO, or 200 μM SHP2 inhibitor (SHP2i) for 1 h and then forskolin for 45 min before treatment with IFN-α/β for 30 min. The SHP2 proteins were co-immunoblotted using SHP2 antibodies from the cell lysates and the activity of SHP2 proteins in each group was detected using microplate reader (excitation, 355 nm; emission, 460 nm) *$p < 0.05$, **$p < 0.01$ vs. IFN-α-treated group, ###$p < 0.001$ vs. IFN-α treated group (unpaired two-tailed Student's *t*-test). *T,* HEK293A cells were pretreated with DMSO, or 200 μM SHP2 inhibitor (SHP2i) for 1 h and then forskolin for 45 min before treatment with IFN-α/β for 30 min. The SHP2 proteins were co-immunoblotted using SHP2 antibodies from the cell lysates and the activity of SHP2 proteins in each group was detected using microplate reader (excitation, 355 nm; emission, 460 nm). *$p < 0.05$, **$p < 0.01$ and ***$p < 0.001$ vs. IFN-α/β-treated group, ###$p < 0.001$ vs. IFN-α/β + forskolin-treated group (unpaired two-tailed Student's *t*-test). IFN-α, 5000 U/mL; IFN-β,1000 U/mL; Forskolin, 50 μM; PKA catalytic subunit, 25 U/μL. All experiments were conducted with three independent replicates and the results of representative data are shown. The data are presented as the mean ± SD from three independent experiments.

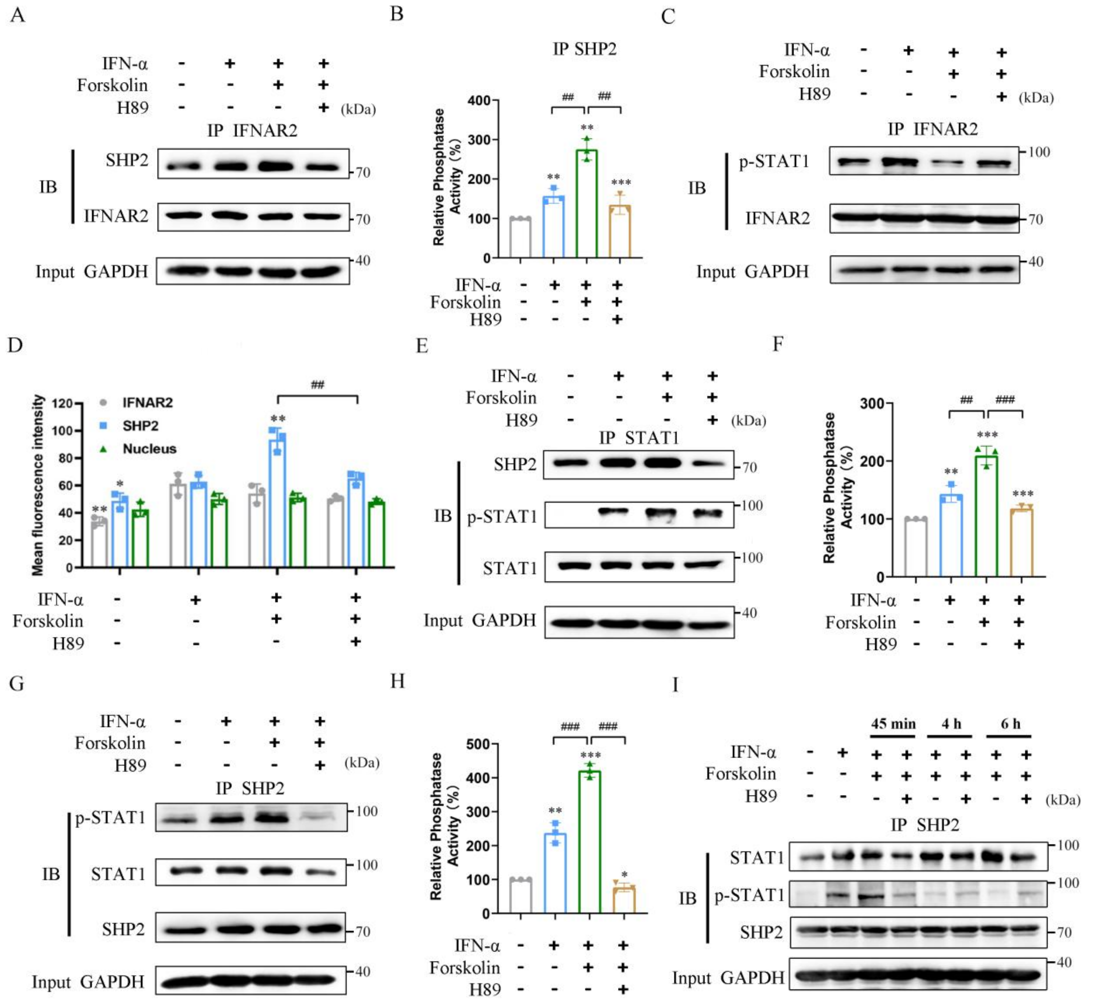

Fig. S2 PKA promotes SHP2 interaction with STAT1. *A, B,* Huh-7 cells were pretreated with DMSO or H89 for 1 h and forskolin for 45 min before treatment with IFN-α for 30 min. Cell lysates were immunoprecipitated with IFNAR2 antibody, and the co-immunoprecipitation (co-immunoprecipitation) products were divided into two parts. (*A*) One sample was immunoblotted with an SHP2 antibody. IFNAR2 was used as a loading control. (*B*) Another sample was detected in another phosphatase activity of SHP2. **p < 0.01, ***p < 0.001 vs. control group, ##p < 0.01 vs. IFN-α + forskolin treatment group (unpaired two-tailed Student's *t*-test). *C,* Huh-7 cells were pretreated with DMSO or H89 for 1 h and forskolin for 45 min before treatment with IFN-α for 30 min. Cell lysates were immunoprecipitated with IFNAR2 antibody, and the co-immunoprecipitation products were immunoblotted with phospho-STAT1 (Tyr701)

antibodies. IFNAR2 was used as a loading control. *D,* The mean fluorescence intensity of IFNAR2, SHP2 in HEK293A cells. *$p < 0.05$ and **$p < 0.01$ vs. IFN-α-treated group. ##$p < 0.01$ vs. IFN-α + forskolin-treated group (unpaired two-tailed Student's *t*-test). *E, F,* Huh-7 cells were pretreated with DMSO or H89 for 1 h and forskolin for 45 min before treatment with IFN-α for 30 min. Cell lysates were immunoprecipitated with STAT1 antibody, and the co-immunoprecipitation products were divided into two parts. One sample was immunoblotted with SHP2 and phospho-STAT1 (Tyr701) antibodies. STAT1 was used as a loading control (*E*). Another sample was used to detect the phosphatase activity of SHP2 (*F*). **$p < 0.01$, ***$p < 0.001$ vs. control group, ##$p < 0.01$, ###$p < 0.001$ vs. IFN-α + forskolin treatment group (unpaired two-tailed Student's *t*-test). *G, H,* Huh-7 cells were pretreated with DMSO or H89 for 1 h and forskolin for 45 min before treatment with IFN-α for 30 min. Cell lysates were immunoprecipitated with SHP2 antibody, and the co-immunoprecipitation products were divided into two parts. One sample was immunoblotted using antibodies against STAT1 and phospho-STAT1 (Tyr701). SHP2 antibody staining was used as a loading control (*G*). The other sample was used to detect the phosphatase activity of SHP2 (*H*). *$p < 0.05$, **$p < 0.01$, ***$p < 0.001$ vs. control group, ###$p < 0.001$ vs. IFN-α + forskolin treatment group (unpaired two-tailed Student's *t*-test). *I,* Huh-7 cells were pretreated with DMSO or H89 for 1 h, followed by forskolin treatment for 45 min, 4 h, and 6 h, respectively, before being treated with IFN-α for 30 min. Cell lysates were immunoprecipitated using an SHP2 antibody, and the co-immunoprecipitation products were immunoblotted with antibodies against STAT1 and phospho-STAT1 (Tyr701). SHP2 was used as a loading control. All experiments were conducted with three independent replicates and the results of representative data are shown. The data are presented as the mean ± SD from three independent experiments.

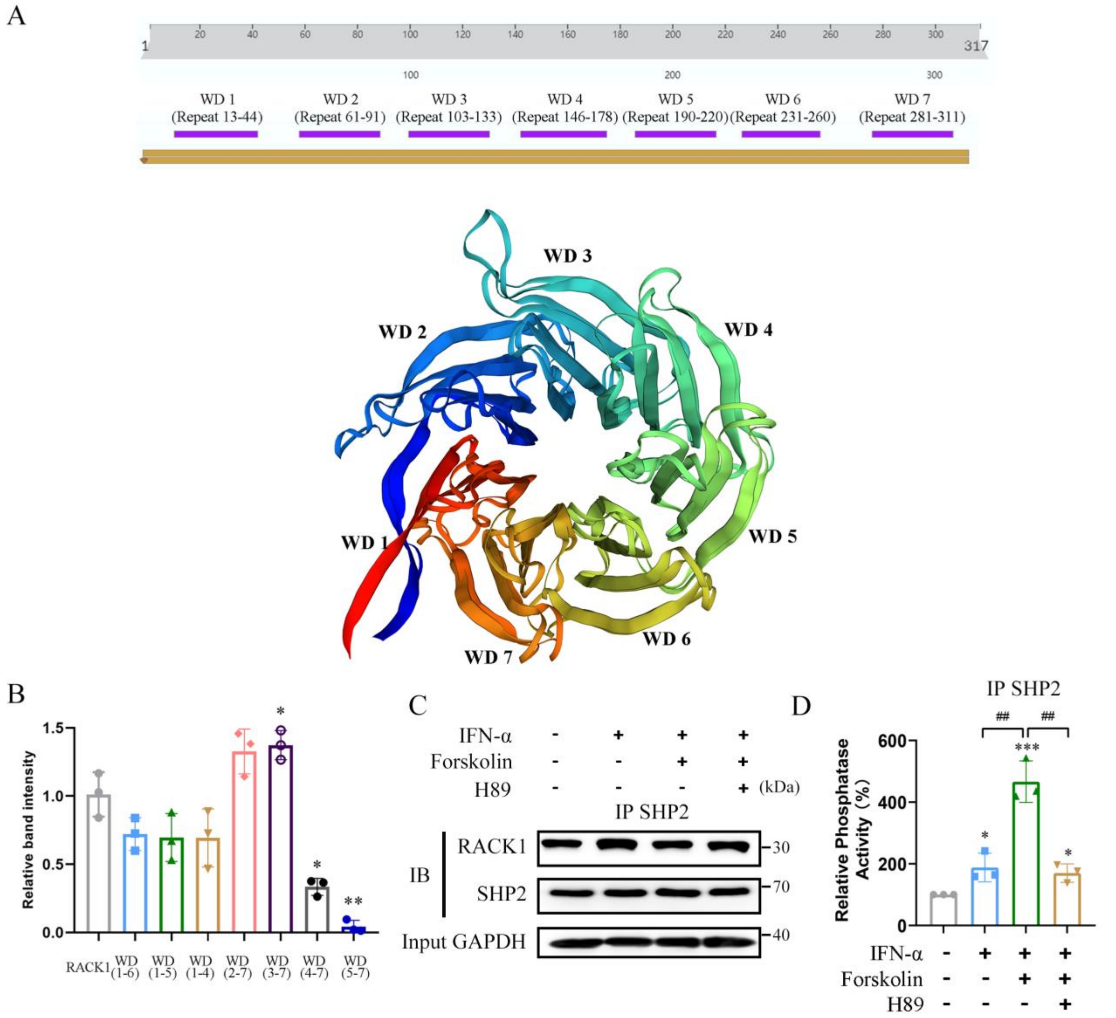

Fig. S3 PKA promotes the dissociation of SHP2 from RACK1. *A,* Diagram of RACK1 containing WD domains. *B,* Quantification of the PKARIIlevels in the western blotting analysis (Fig. 3B) was performed, with protein levels normalized to GST and expressed as relative values. *p < 0.05, **p < 0.01 vs. RACK1 group (unpaired two-tailed Student's *t*-test). *C, D,* Huh-7 cells were pretreated with DMSO or forskolin for 45 min. After that the cells were treated with IFN-α for 30 min. Cell lysates were immunoprecipitated with the RACK1 antibody, and the co-immunoprecipitation products were divided into two parts. One sample was immunoblotted using an SHP2 antibody. RACK1 antibody was used as a loading control (*C*). The other sample was used to detect the phosphatase activity of SHP2 (*D*). *p < 0.05, ***p < 0.001 vs. control group, ##p < 0.01 vs. IFN-α + forskolin treatment group (unpaired two-tailed Student's *t*-test). All

experiments were conducted with three independent replicates and the results of representative data are shown. The data are presented as the mean ± SD from three independent experiments.

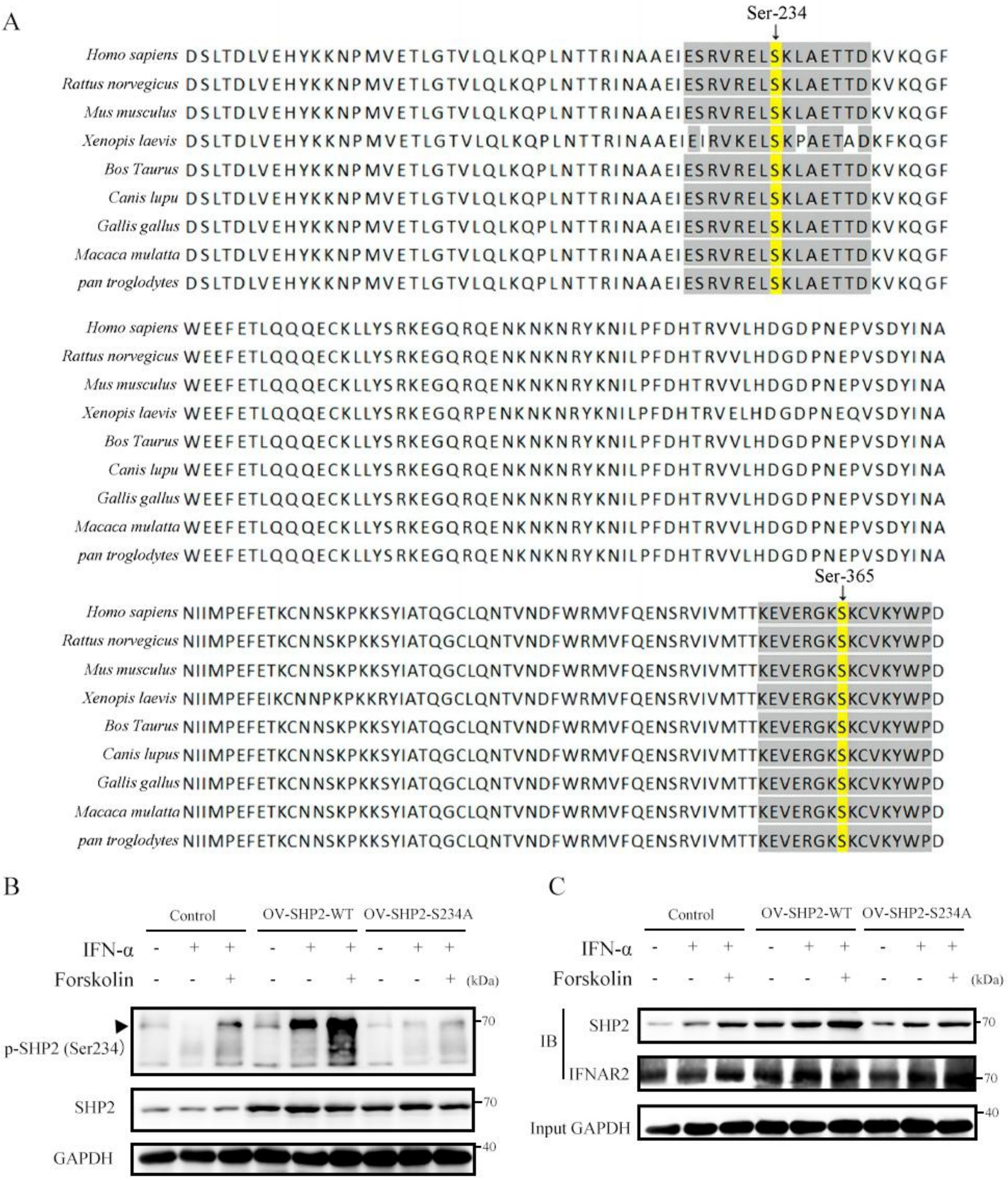

Fig. S4 PKA phosphorylates SHP2 and promotes its activity. *A,* Comparison of SHP2 protein sequences from different species. *B,* Huh-7 cells transiently transfected with pCMV-SHP2 or pCMV-SHP2^{S234A}. After 48 h, the cells were pretreated with DMSO or forskolin for 4 h, and then treated with IFN-α for 30 min. Cell lysates were immunoprecipitated with SHP2 antibody, and the co-immunoprecipitation (Co-IP) products were immunoblotted with an p-SHP2 (Ser234) antibody. GAPDH was used as a loading control. *C,* Huh-7 cells transiently transfected with pCMV-SHP2

or pCMV-SHP2^{S234A}. After 48 h, the cells were pretreated with DMSO or forskolin for 4 h, and then treated with IFNα for 30 min. Cell lysates were immunoprecipitated with IFNAR2 antibody, and the co-immunoprecipitation (Co-IP) products were immunoblotted with an SHP2 antibody. GAPDH was used as a loading control. All experiments were conducted with three independent replicates and the results of representative data are shown.

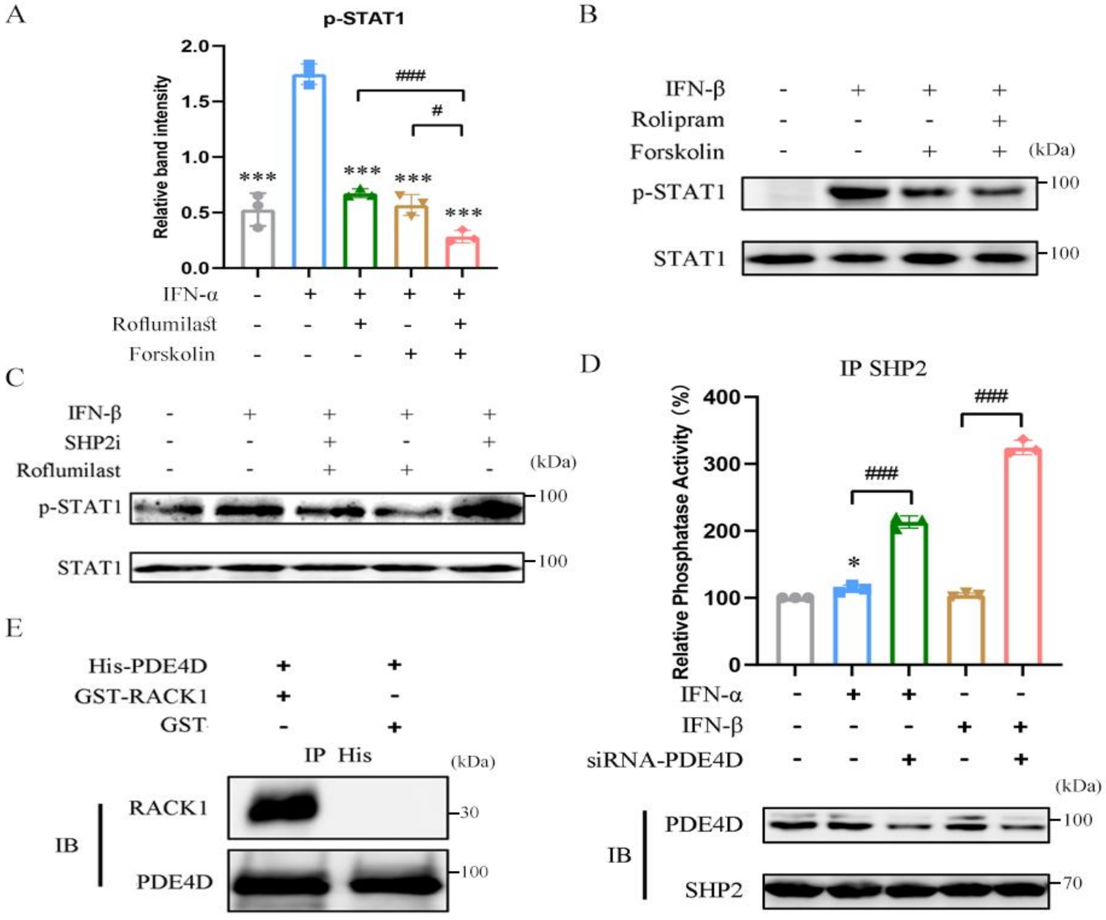

Fig. S5 PDE4D regulates SHP2 activity via RACK1. *A,* Quantification of the p-STAT1 levels in the western blotting analysis (Fig. 5B) was performed, with protein levels normalized to STAT1 and expressed as relative values. ***p < 0.001 vs. IFN-α-treated group, #p < 0.05 vs. IFN-α + forskolin-treated group, ###p < 0.001 vs. IFN-α + Roflumilast-treated group (unpaired two-tailed Student's t-test). *B,* HEK293A cells were pretreated with DMSO or 10 μM Rolipram for 1 h and then with 50 μM forskolin for 45 min before treatment with IFN-β for 30 min. Cell lysates were immunoblotted using antibodies against phospho-STAT1 (Tyr701) and STAT1. *C,* HEK293A cells were pretreated with DMSO or 1 μM Roflumilast or 200 μM SHP2 inhibitor (SHP2i) for 1 h before treatment with IFN-β for 30 min. Cell lysates were immunoblotted using antibodies against phospho-STAT1 (Tyr701) and STAT1. *D,* HEK293A cells transfected with siRNAcon (50 nM) or

PDE4D siRNA001 (50 nM). After 72 h, the cells were treated with IFN-α/β for 30 min. Cell lysates were immunoprecipitated with an SHP2-specific antibody against total SHP2. Total SHP2 immunoprecipitates were divided into two parts for the SHP2 activity assay and for western blotting as a loading control. *p< 0.05 vs. control treated group, ###p < 0.001 vs. IFN-α/β treated group (unpaired two-tailed Student's *t*-test). *E,* The purified recombinant GST-RACK1 and His-PDE4D were incubated *in vitro*, and the samples were separated and detected using RACK1 and PDE4D antibodies. IFN-α, 5000 U/mL; IFN-β,1000 U/mL. All experiments were conducted with three independent replicates and the results of representative data are shown. The data are presented as the mean ± SD from three independent experiments.

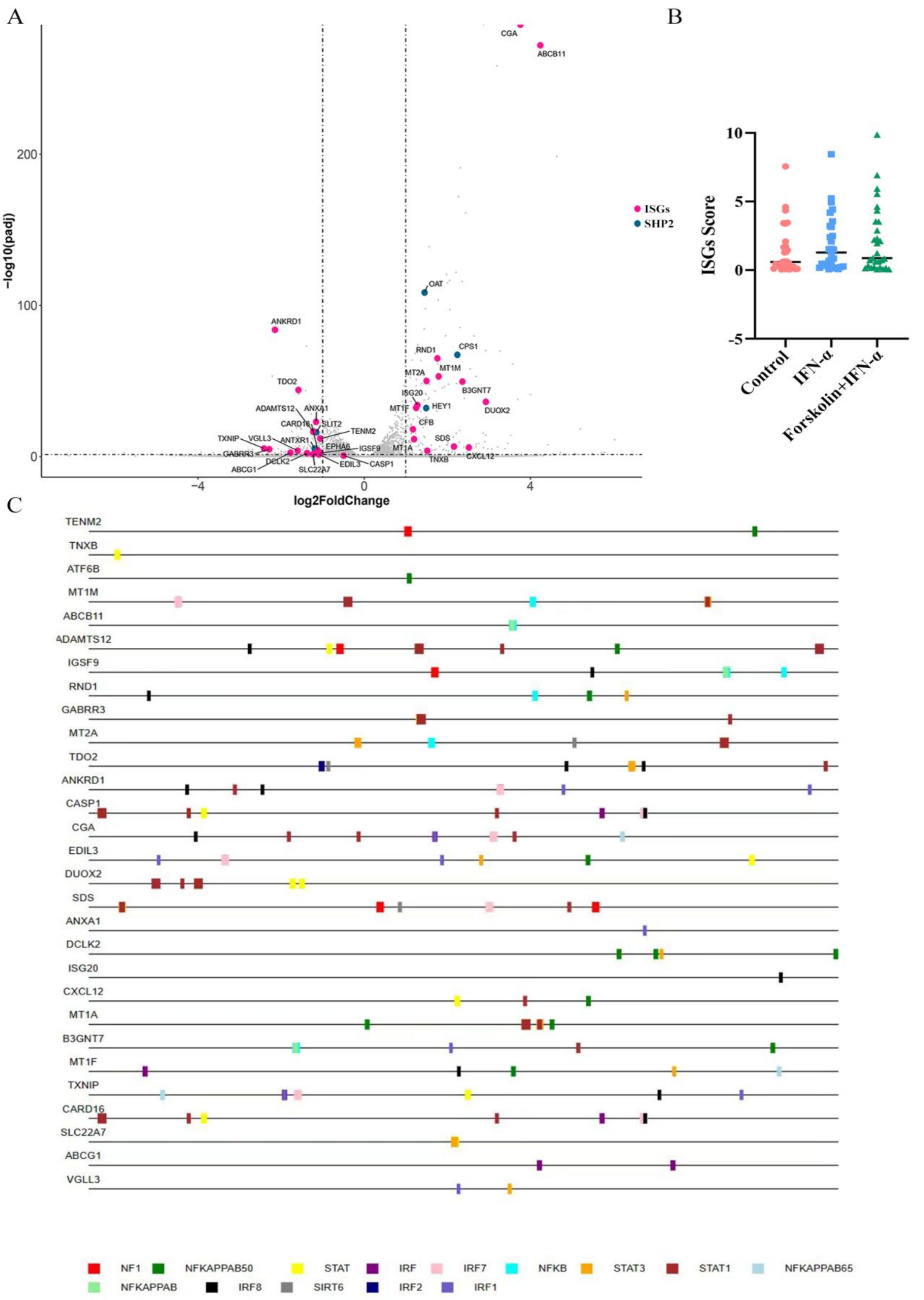

Fig. S6 Transcriptome analysis of PKA activation in HCC cells. *A,* ISGs and SHP2 signature transcriptional changes in the IFN-α and forskolin + IFN-α groups. *B,* ISGs score in Control, IFN-α and forskolin + IFN-α groups. The gene signature score was calculated using the

normalized $\log_2$ ($N_{gene\ expression\ data}$+1) from 29 ISGs. *C,* A list of transcription factors (TFs) of the 29 differentially expressed ISGs was obtained based on the interferome database. (n = 3, each group).

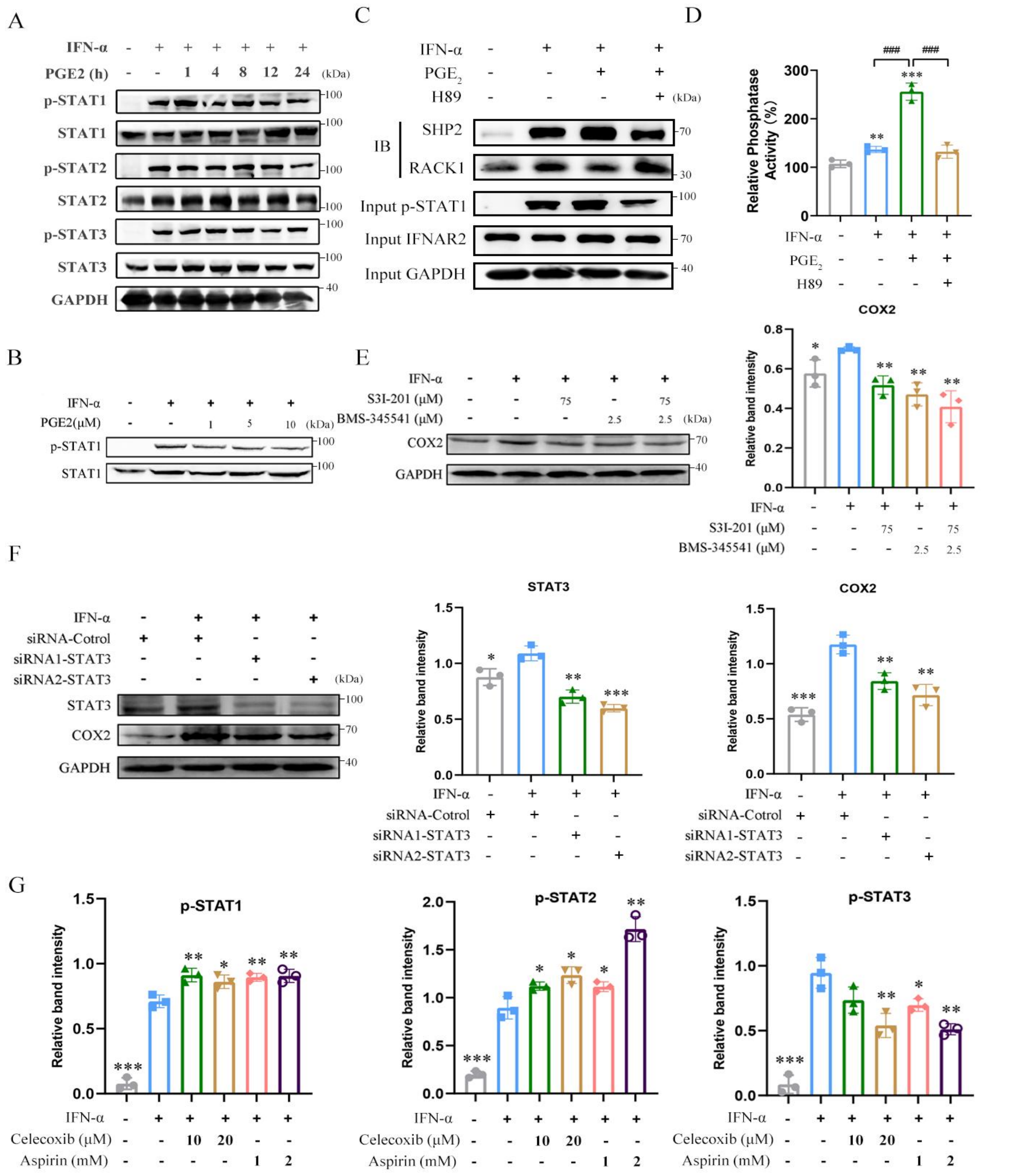

Fig. S7 PGE_2 suppresses activation of JAK/STAT through PKA/SHP2 signaling. *A,* HCCLM3 cells were pretreated with DMSO or 50 μM PGE_2 for the indicated time, and then treated with IFN-α for 30 min. Cell lysates were immunoblotted with antibodies against phospho-STAT1 (Tyr701), phospho-STAT2 (Tyr690), phospho-STAT3 (Tyr705), STAT1, STAT2 and STAT3 antibodies. *B,* HEK293A cells were pretreated with PGE_2 in dose-dependent manner for 10 min before treatment with IFN-α for 30 min. Cell lysates were immunoblotted for phospho-STAT1

(Tyr701). STAT1 staining was used as a loading control. *C, D,* Cell lysates were immunoprecipitated with the IFNAR2 antibody, and the co-immunoprecipitation (Co-IP) products were divided into two parts. One sample was immunoblotted using SHP2 and RACK1 antibodies. In addition, 5% of the cell lysates were immunoblotted using antibodies against phospho-STAT1 (Tyr701) and IFNAR2. GAPDH staining was used as a loading control (*C*). The other sample was used to detect the phosphatase activity of SHP2 (*D*). **$p < 0.01$, ***$p < 0.001$ vs. control group, ###$p < 0.01$ vs. IFN-α + PGE_2 treatment group (unpaired two-tailed Student's *t*-test). *E,* HEK293A cells were pretreated with DMSO or 75 μM S3I-201 or 2.5 μM BMS-345541 for 1 h before treatment with IFN-α for 30 min. Cell lysates were immunoblotted with COX2 antibody. Quantification of the COX2 levels in the western blotting analysis was performed, with protein levels normalized to GAPDH and expressed as relative values. *$p < 0.05$, **$p < 0.01$ vs. IFN-α-treated group (unpaired two-tailed Student's *t*-test). *F,* HEK293A cells were transfected with control (50 nM), siRNA1-STAT3 (50 nM), or siRNA2-STAT3 (50 nM). After 72 h, the cells were incubated with IFN-α for 30 min. Cell lysates were immunoblotted with STAT3, COX2, and GAPDH. Quantification of the COX2 levels in the western blotting analysis was performed, with protein levels normalized to GAPDH and expressed as relative values. *$p < 0.05$, **$p < 0.01$ and ***$p < 0.001$ vs. IFN-α-treated group (unpaired two-tailed Student's *t*-test). *G,* Quantification of the p-STAT1/2/3 levels in the western blotting analysis (Fig. 7G) was performed, with protein levels normalized to STAT1/2/3 and expressed as relative values. *$p < 0.05$, **$p < 0.01$ and ***$p < 0.001$ vs. IFN-α-treated group. IFN-α, 5000 U/mL. All experiments were conducted with three independent replicates and the results of representative data are shown. The data are presented as the mean ± SD from three independent experiments.

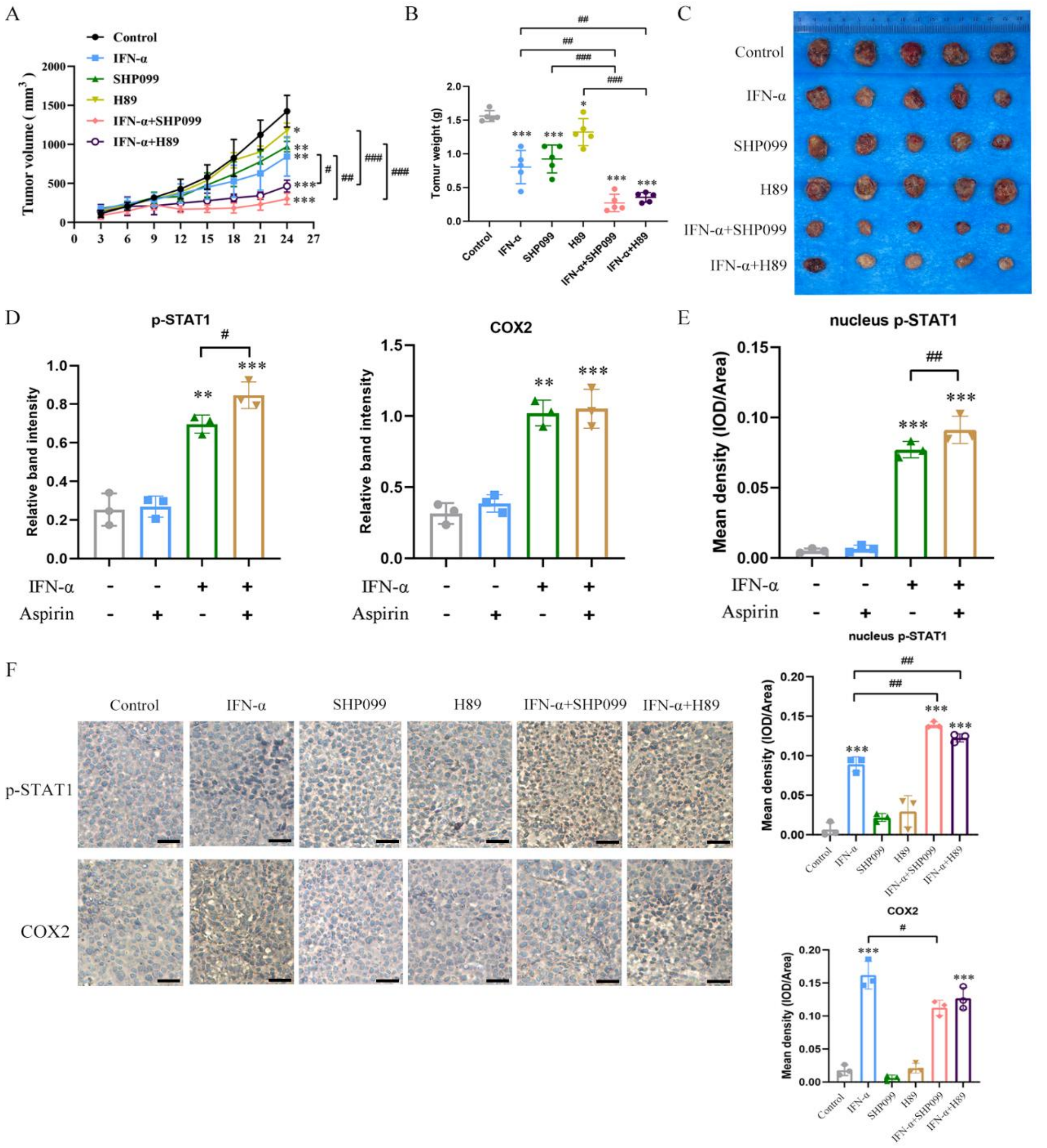

Fig. S8 Aspirin promotes the antiproliferative effect of IFN-α *in vivo*. *A-C,* Xenograft tumor (Huh-7 cell) mice were treated with SHP099, H89 or IFN-α plus SHP099/H89 for 24 days and the tumor growth, tumor weight and representative images are shown. (n = 6, each group). *D,* Quantification of the p-STAT1 and COX2 levels in the western blotting analysis (Fig. 8D) was performed, with protein levels normalized to GAPDH and expressed as relative values. ***p* < 0.01, ****p* < 0.001 vs. control group, #*p* < 0.05 vs. IFN-αtreated group (unpaired two-tailed Student's *t*-test). *E,* The level of phosphorylated STAT1 in the nucleus was quantified. ****p* < 0.001 vs.

control group, ##$p < 0.01$ vs. IFN-α treatment group (unpaired two-tailed Student's *t*-test). IFN-α, 1× 10^5 U/kg; Aspirin, 15 mg/kg. *F,* The phosphorylation of STAT1 and expression of COX2 in tumor tissues were detected by immunohistochemistry (Scale bar = 20 μm). The level of phosphorylated STAT1 in the nucleus and COX2 were quantified. ***$p < 0.001$ vs. control group, #$p < 0.05$, ##$p < 0.01$ vs. IFN-α treatment group (unpaired two-tailed Student's *t*-test). IFN-α, 1× 10^5 U/kg; SHP099, 10 mg/kg; H89, 10 mg/kg. All experiments were conducted with three independent replicates and the results of representative data are shown. The data are presented as the mean ± SD from three independent experiments.

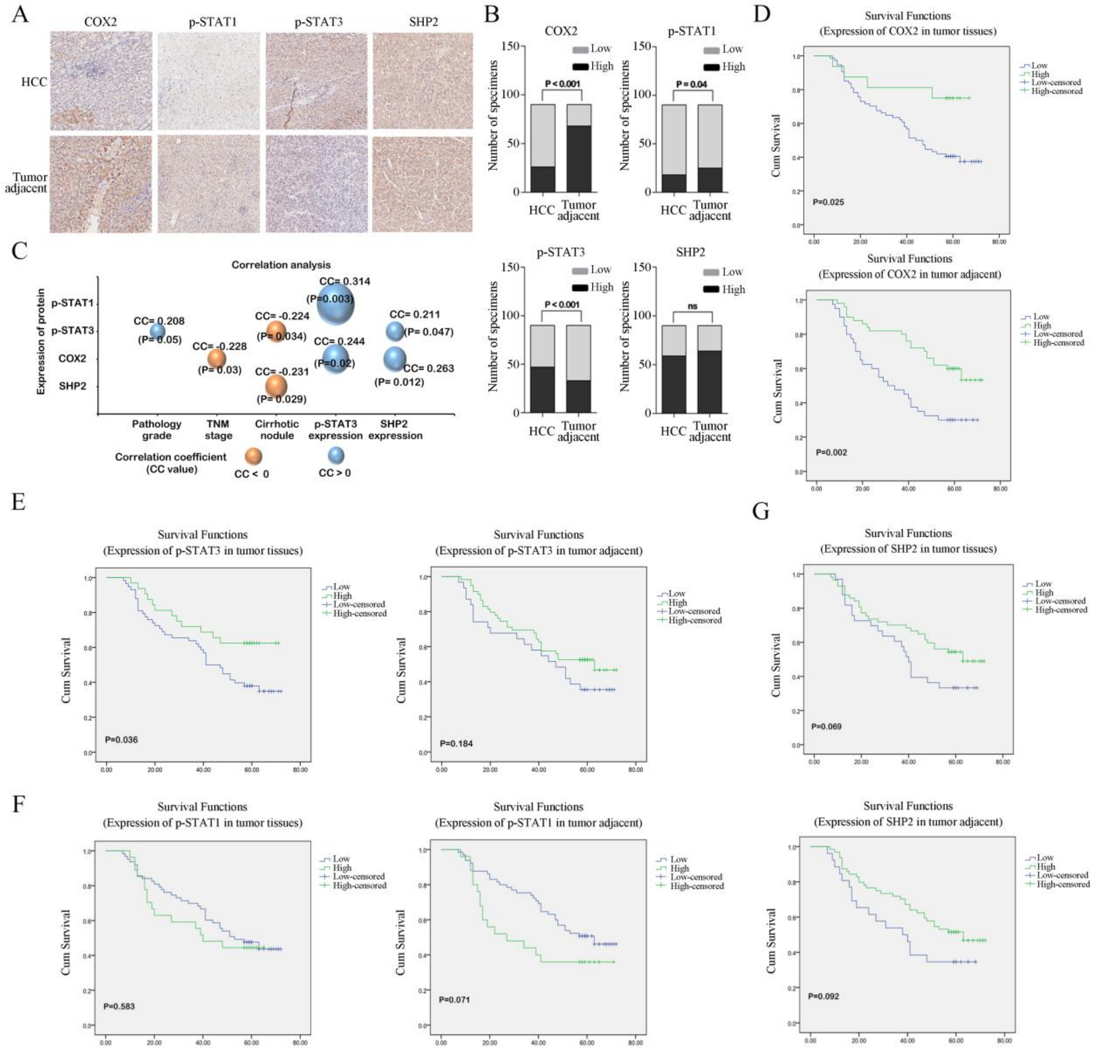

Fig. S9 Correlations between STAT1/3-SHP2-COX2 axis expression and human HCC development. *A,* Tissue microarrays were used to analyze the phosphorylation of STAT1/3 and the expressions of COX2 and SHP2 in patients with HCC. (n = 90). *B,* Bar chart representing the quantitative statistics of COX2, p-STAT1, nuclear p-STAT3, and SHP2 expression in tissues of patients with HCC. *C,* Correlation analysis between protein expression levels (p-STAT1/3, COX2, SHP2), pathological grade, and TNM grade. The highest CC value was 1, indicating a perfect positive correlation (blue), and the lowest CC value was -1, indicating a perfect negative correlation (orange). *D, E,* Survival curves showing a strong correlation between expression levels (COX2 and p-STAT3) and patient prognosis. Survival analyses were performed using the

Kaplan-Meier method. *F, G,* Kaplan–Meier curves showing no correlations between p-STAT1 level, SHP2 expression and prognosis.
